# Site-directed conjugation of single-stranded DNA to affinity proteins: quantifying the importance of conjugation strategy†

**DOI:** 10.1039/d4sc01838a

**Published:** 2024-05-07

**Authors:** Andres Rocha Tapia, Fabrice Abgottspon, Johan Nilvebrant, Per-Åke Nygren, Sarah Duclos Ivetich, Andres Javier Bello Hernandez, Ioanna A. Thanasi, Peter A. Szijj, Ghali Sekkat, François M. Cuenot, Vijay Chudasama, Nicola Aceto, Andrew J. deMello, Daniel A. Richards

**Affiliations:** a Institute for Chemical and Bioengineering, ETH Zurich Vladimir-Prelog-Weg 1 8093 Zürich Switzerland daniel.richards@chem.ethz.ch; b Department of Protein Science, KTH Royal Institute of Technology, AlbaNova University Center 106 91 Stockholm Sweden; c Department of Chemistry, University College London 20 Gordon Street WC1H 0AJ London UK; d Department of Biology, Institute of Molecular Health Sciences, ETH Zurich Otto-Stern-Weg 7 8093 Zürich Switzerland

## Abstract

Affinity protein–oligonucleotide conjugates are increasingly being explored as diagnostic and therapeutic tools. Despite growing interest, these probes are typically constructed using outdated, non-selective chemistries, and little has been done to investigate how conjugation to oligonucleotides influences the function of affinity proteins. Herein, we report a novel site-selective conjugation method for furnishing affinity protein–oligonucleotide conjugates in a 93% yield within fifteen minutes. Using SPR, we explore how the choice of affinity protein, conjugation strategy, and DNA length impact target binding and reveal the deleterious effects of non-specific conjugation methods. Furthermore, we show that these adverse effects can be minimised by employing our site-selective conjugation strategy, leading to improved performance in an immuno-PCR assay. Finally, we investigate the interactions between affinity protein–oligonucleotide conjugates and live cells, demonstrating the benefits of site-selective conjugation. This work provides critical insight into the importance of conjugation strategy when constructing affinity protein–oligonucleotide conjugates.

## Abstract 2

Site-selective conjugation of ssDNA to affinity proteins grants improved target binding and lower non-specific binding.

## Graphical abstract

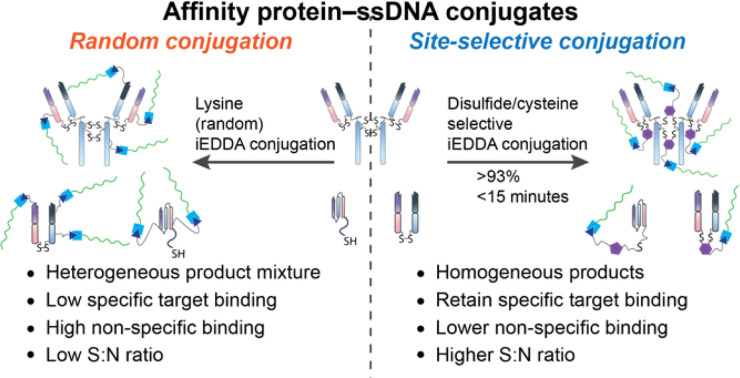

## Introduction

Over the last two decades, affinity protein–oligonucleotide conjugates have become indispensable tools within analytical and diagnostic assays^1^ and are increasingly being explored as therapeutic agents.^2^ Whilst the applications of affinity protein–oligonucleotide conjugates are growing steadily, the methods we use to generate them remain largely unchanged. This is in spite of a rapidly expanding toolbox of chemical transformations designed to facilitate the conjugation of affinity proteins to a plethora of chemical and biological moieties.^3^ Furthermore, there is a paucity of studies assessing the relative influence of conjugation strategy, as well as protein and oligonucleotide structure, on the performance of affinity protein–oligonucleotides. As a result, we possess an inadequate understanding of the factors underpinning both the construction and application of these important bioconjugates. In this study, we set out to remedy this by investigating the impact of both conjugation strategy and protein/oligonucleotide structure on the binding properties of a selection of affinity protein–oligonucleotide conjugates.

Presently, affinity protein–oligonucleotide conjugates typically comprise IgGs conjugated to single-stranded DNA/RNA *via* surface-accessible lysine residues, either directly *via* covalent chemistries^2,4–8^ or indirectly *via* non-covalent ionic/affinity-based interactions.^9^ These approaches take advantage of the simplicity of lysine conjugation. Reagents such as NHS-esters and isothiocyanates are easy to synthesise, and also readily available from commercial suppliers (as are IgGs).^5–8^ Though convenient, covalent conjugation to lysine residues has a significant disadvantage; lysine residues are typically abundant on protein surfaces and almost impossible to selectively target on native proteins. Consequently, such an approach results in highly heterogeneous bioconjugate mixtures and can significantly impact target binding.^7,9^ Heterogeneity in affinity protein-based targeting ligands is associated with a plethora of issues, including decreased target affinity, batch-to-batch variability, increased non-specific binding, poor stability, and unpredictable pharmacokinetic properties.^10^ Furthermore, a reliance on large IgGs is problematic for proximity-driven biosensing assays, such as proximity-ligation assays (PLA) and super-resolution imaging (*e.g.*, DNA Points Accumulation for Imaging in Nanoscale Topography: DNA-PAINT), where minimising the distance between the oligonucleotide probe and the target is paramount.^11–14^

Understanding these limitations, researchers have begun constructing affinity protein–oligonucleotide conjugates using more controlled conjugation chemistries and/or non-IgG affinity proteins, or smaller IgG-derived ligands. To date, site-selective conjugation of oligonucleotides to IgGs has been achieved *via* reduced disulphide bonds,^15^ specifically engineered cysteine or lysine residues,^16,17^ transglutaminase/sortase-mediated enzymatic conjugation,^18^ or by employing DNA-templated protein conjugation (DTPC).^1^ Similar strategies have been used to conjugate oligonucleotides to IgG-derived affinity proteins such as Fab^19^ and scFv^13^ ligands. Though there are many reported advantages of using non-IgG-derived affinity binders,^20^ particularly for analytical applications, their conjugation to oligonucleotides remains underexplored. Camelid-based nanobody–oligonucleotide conjugates have carved a niche as probes in DNA-PAINT assays,^11,21–23^ PLA,^24,25^ and proximity extension assays (PEA).^26^ Designed Ankyrin Repeat Proteins (DARPins) have been similarly employed for PLA and immuno-rolling circle amplification (iRCA) assays.^27^ Though the field is nascent,^28^ monobodies (centyrins),^29^ nanobodies,^30^ and DARPins^31^ have all been conjugated to siRNA for therapeutic applications. Unfortunately, and despite the established benefits of using these synthetic scaffolds, IgGs remain the *de facto* ligand choice for creating affinity protein–oligonucleotides.

A common feature of the aforementioned studies is that they have largely failed to provide detailed comparisons between site-specific conjugation and traditional non-selective lysine conjugation, or non-IgG ligands and IgG ligands. Particularly lacking are investigations into how changing the nature of the affinity ligand and the oligonucleotide conjugation strategy influence both specific and non-specific binding properties of affinity proteins. A lone study by Lehot *et al.* investigated the non-specific interactions of IgG–DNA conjugates with mammalian cells, concluding that ssDNA conjugates exhibited far greater non-specific binding to SK-BR-3 and MDA-MB-231 cells when compared to dsDNA conjugates.^32^ The authors did not explore different conjugation methods or affinity ligands or draw any firm conclusions regarding the effect of conjugation on specific binding. To date, no study of this type exists.

Given that oligonucleotides are complex, negatively charged molecules capable of engaging in multiple non-covalent interactions, it is reasonable to hypothesise that their conjugation to affinity protein ligands could influence ligand–target interactions. This hypothesis can be extended to propose that the location and degree of conjugation could influence the magnitude of these effects. In this study, we set out to test this hypothesis by systematically investigating the impact of oligonucleotide conjugation strategy on the binding profiles and analytical performance of multiple affinity protein–oligonucleotide conjugates. We combined non-selective and site-selective chemistries with IgG and non-IgG ligands to construct a library of affinity protein–oligonucleotide conjugates and analysed their target binding profiles using surface plasmon resonance. We then employed these affinity protein–oligonucleotide conjugates in a plate-based immuno-PCR assay and as probes for cell-surface receptor imaging, at each stage comparing and contrasting the different approaches. The present study thus contributes to our understanding of this important class of bioconjugate and creates a much clearer picture of how they ideally should be constructed.

## Results and discussion

### Protein–ssDNA conjugation

**Fig. 1:**
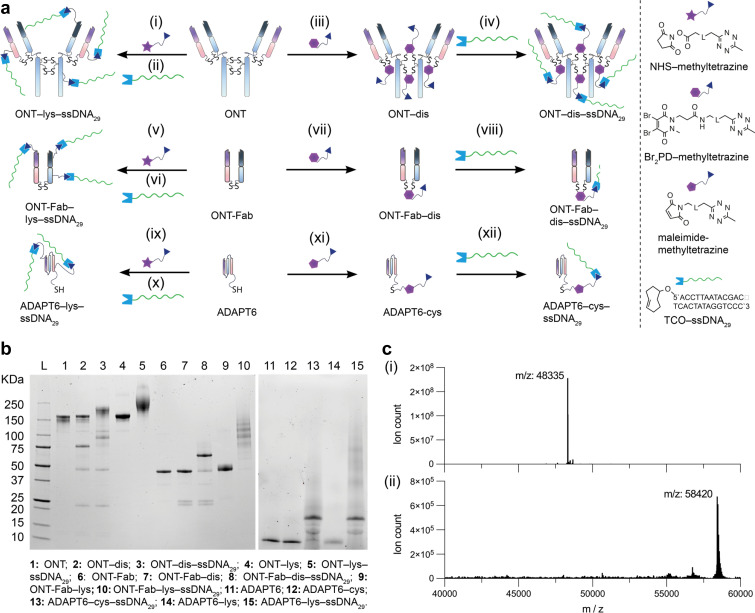
Site-selective iEDDA click chemistry is an efficient method for constructing affinity protein–ssDNA conjugates. (a) Schematic representations of the modification of Ontruzant (ONT), Ontruzant Fab (ONT-Fab), and ADAPT6. The modification conditions are: (i) NHS–methyltetrazine, 10 eq., 37 °C, 2 h, BBS pH = 8.4. (ii) ssDNA_29_, 8 eq., 21 °C, 0.5 h, BBS pH = 8.0. (iii) TCEP·HCl, 40 eq., 37 °C, 2 h, BBS pH = 8.0, Br_2_PD–methyltetrazine, 24 eq., 21 °C, 1.5 h, BBS pH = 8.0. (iv) ssDNA_29_–TCO, 10 eq., 21 °C, 0.5 h, BBS pH = 8.0. (v) NHS–methyltetrazine, 10 eq., 21 °C, 2 h, BBS pH = 8.4. (vi) ssDNA_29_–TCO, 6 eq., 21 °C, 0.5 h, BBS pH = 8.0. (vii) TCEP·HCl, 10 eq., 37 °C, 2 h, BBS pH = 8.0, Br_2_PD–methyltetrazine, 8 eq., 21 °C, 1.5 h, BBS pH = 8.0. (viii) ssDNA_29_–TCO, 6 eq., 21 °C, 0.5 h, BBS pH = 8.0. (ix) NHS–methyltetrazine, 10 eq., 21 °C, 2 h, BBS pH = 8.4. (x) ssDNA_29_, 6 eq., 21 °C, 0.5 h, BBS pH = 8.0. (xi) Maleimide–methyltetrazine, 10 eq., 21 °C, 1.5 h, BBS pH = 8.0. (xii) ssDNA_29_–TCO, 6 eq., 21 °C, 0.5 h, BBS pH = 8.0. Full chemical structures of Br_2_PD–methyltetrazine, maleimidemethyltetrazine, and NHS–methyltetrazine can be found in Table S1.† (b) SDS-PAGE analysis of the modified ONT, ONT-Fab, and ADAPT6 structures. Lanes 11–15 were run on a separate gel. Lanes 3 and 8 highlight the advantages of site-directed conjugation for creating highly homogenous affinity protein–ssDNA conjugates. (c) Deconvoluted mass spectra of (i) ONT-Fab–dis and (ii) ONT-Fab–dis–ssDNA_29_. More detailed spectra can be found in Fig. S2.† The presence of a single product confirms the disulfide-selective nature of the reaction between ONT–dis and ssDNA_29_.

To begin, we established methodologies for installing complementary reactive “click” handles onto both the affinity proteins and the ssDNA oligonucleotides. Due to the fast reaction kinetics, we opted to employ the inverse electron demand Diels–Alder (iEDDA) reaction between 1,2,4,5-methyltetrazine and *trans*-cyclooctene. As model proteins, we chose Ontruzant (trastuzumab, ONT), the Fab fragment of Ontruzant (ONT-Fab), and an ADAPT6 equipped with a unique N-terminal cysteine residue (ADAPT6).^33^ ADAPT6 is a scaffold affinity protein based on the albumin binding domain of streptococcal protein G. These proteins all target the same extracellular epitope on domain IV of human epidermal growth factor 2 (HER2)^34^ but vary significantly in their size (*ca.* 7–145 kDa). We installed methyltetrazine handles onto the ONT and ONT-Fab using established disulfide-bridging dibromopyridazinedione chemistry (Fig. 1a and S1a–j,† conjugates denoted as “dis”).^35^ In the case of the ADAPT6–cys, we employed maleimide chemistry to install the methyltetrazine onto the N-terminal cysteine (conjugate denoted as “cys”). These sites (disulfide bridges and N-terminal cysteine) were chosen due to their distance from the HER2-targeting paratope. We hypothesised that maximising the distance between the conjugation site and the paratope would lead to more efficient target binding. To enable comparison with non-selective chemistries, we also installed methyltetrazine handles onto random lysine residues within each ligand using NHS-ester chemistry (conjugates denoted as “lys”). Successful installation of the click handles was evidenced using SDS-PAGE (Fig. 1b) and LC-MS (Fig. 1c and S2†). In the case of the site-selective modification of ONT and ONT-Fab, partial rebridging was observed, as evidenced by the presence of lower molecular weight bands on the SDS-PAGE (Fig. 1b, lanes 2, 3, 7, 8). However, densitometry analysis suggests the desired, modified proteins account for >90% of the material in the sample. To enable conjugation between the methyltetrazine-modified affinity and ssDNA, we installed *trans*-cyclooctene handles onto ssDNA using a bifunctional DBCO–PEG_12_–TCO linker and azide-modified oligos (Fig. S3 and Table S2†). Following this, we optimised the conjugation of a TCO-functionalized 29 nucleotide ssDNA oligonucleotide (TCO–ssDNA_29_) to each of the affinity proteins (Fig. 1a–c). In each case, we observed quantitative or near-quantitative conversion to the desired product using a 2–3-fold excess of TCO–ssDNA_29_ over the methyltetrazine handles. We subsequently determined that the reaction between ONT-Fab–dis and TCO–ssDNA_29_ is complete in as little as 15 minutes using just three equivalents of TCO–ssDNA_29_, with a conversion rate >93% (Fig. S4†). We attribute the high efficiency of ssDNA conjugation to the fast kinetics of the iEDDA reaction (1–10^6^ M^−1^ s^−1^).^36^ Previous “click” approaches for conjugating proteins to ssDNA have typically relied on slower strain-promoted alkyne–azide cycloaddition (SPAAC) chemistries with low reported conversions.^7,37,38^ Purification of ONT–ssDNA_29_ and ONT-Fab–ssDNA_29_ could be achieved *via* anion exchange chromatography.

ADAPT–cys–ssDNA_29_ could not be satisfactorily purified; thus, leftover TCO-modified ssDNA_29_ can be observed in the SDS-PAGE gel (Fig. 1b, lanes 13, 15).

Densitometry analysis of the SDS-PAGE traces of ONT–lys–ssDNA_29_, ONT-Fab–lys–ssDNA_29_, and ADAPT6–cys–ssDNA_29_ suggests average ssDNA : protein ratios of 3.2 : 1, 3.5 : 1, and 1.2 : 1 respectively (Fig. S5†). Using UV-Vis spectroscopy, we determined the pyridazinedione : antibody ratio (PDAR) of ONT–dis to be 3.3 : 1 (Fig. S6†). Assuming quantitative conversion of all methyltetrazine moieties, this would grant an ssDNA : protein ratio of 3.3 : 1. Reacting ONT-Fab–dis and ADAPT6–cys with TCO–ssDNA_29_ yielded singly modified protein–ssDNA_29_ conjugates (ssDNA : protein = 1 : 1) (Fig. 1b and c). This is unsurprising given the 1 : 1 stoichiometry of the chemistries used to generate the methyltetrazine-modified proteins.

As anticipated, SDS-PAGE analysis of the optimised protocols shows that site-selective conjugation of ssDNA grants more homogenous products when compared to non-selective lysine-selective chemistry. When modifying ONT, we achieved similar average ssDNA : protein ratios using both disulfide-selective and lysine-selective chemistries (3.3 : 1 and 3.2 : 1, respectively). However, for ONT-Fab the ssDNA : protein ratios differed significantly between the disulfide-selective and lysine-selection conjugation chemistries (1 : 1 and 3.5 : 1, respectively). This is due to the limitations imposed by the single accessible disulfide bond present within the Fab fragment. Attempts to decrease the degree of labelling of ONT-Fab–lys led to significant amounts of unmodified protein in the reaction solution (Fig. S7†), which was difficult to remove during purification. Intriguingly, in the case of the ADAPT6 both cysteine-selective and lysine-selective chemistries led to similar ssDNA : protein ratios (1 : 1 and 1.2 : 1, respectively). This suggests that there is a certain degree of selectivity when modifying the ADAPT6, either during the reaction between the native protein and the NHS–methyltetrazine or the tetrazine-modified ADAPT6 and the TCO-modified ssDNA.

In the case of ONT-Fab and ADAPT6, trace amounts of unreacted modified proteins remained after incubation with the TCO–ssDNA_29_. Increasing the reaction time or equivalents of TCO–ssDNA_29_ did not improve conversion (Fig. S4†). We attribute this to the well-documented instability of methyltetrazine.^39^ Regardless, this work represents one of the most efficient conjugation reactions between a protein and DNA reported to date. These results also mark the first time that ssDNA has been conjugated to IgG and Fab fragments using disulfide-selective chemistry.

### The impact of conjugation on HER2 binding

**Fig. 2:**
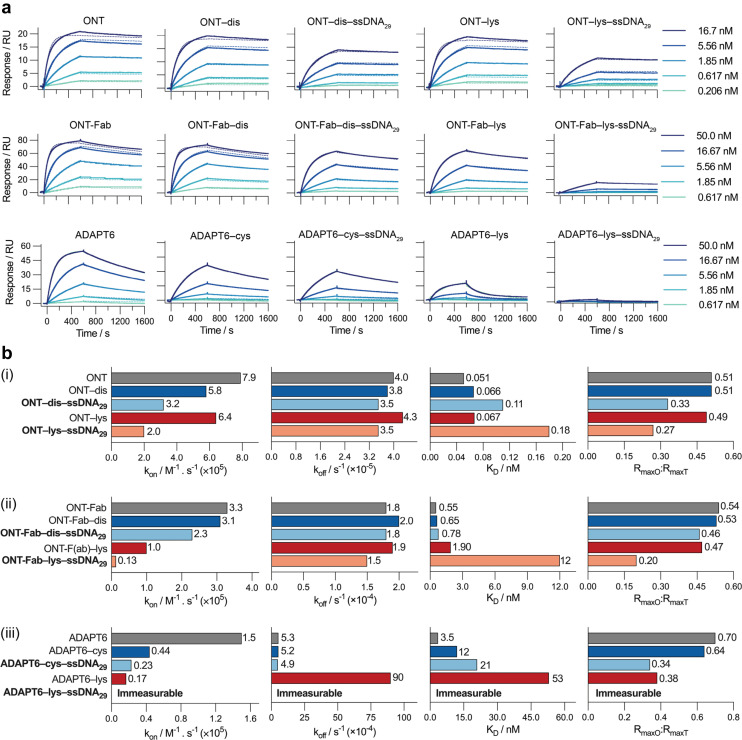
Site-selective conjugation strategies produce bioconjugates with superior target binding. (a) SPR sensorgrams for the binding between HER2 and (top left to bottom right) ONT, ONT–dis, ONT–dis–ssDNA_29_, ONT–lys, ONT–lys–ssDNA_29_, ONT-Fab, ONT-Fab–dis, ONT-Fab–dis–ssDNA_29_, ADAPT6, ADAPT6–cys, ADAPT6–cys–ssDNA_29_, ADAPT6–lys, and ADAPT6–lys–ssDNA_29_. The association binding kinetics were studied over 600 seconds, and the dissociation kinetics over 1000 seconds. Due to the low dissociation rate (*k*_off_), an extended dissociation time (4000 seconds) was employed for studying ONT and its derivatives, as presented in Fig. S8.† Five concentrations were measured for a single sample of each analyte (solid lines), and then globally fit to a 1 : 1 kinetic binding model (dashed lines). (b) Plots of *k*_on_, *k*_off_, *K*_D_, and *R*_maxO_ : *R*_maxT_ for (i) ONT, (ii) ONT-Fab, and (iii) ADAPT6, and their associated bioconjugates. The *k*_on_, *k*_off_, *K*_D_, and *R*_maxO_ values were obtained from a global fit of the data. *R*_maxT_ was determined using eqn (1). The results are summarised in Table S3.† Comparing the ligands conjugated to ssDNA_29_ (bolded) clearly demonstrates that site-selective conjugation leads to higher *k*_on_, lower *K*_D_, and higher *R*_maxO_ : *R*_maxT_.

After successfully preparing the affinity protein–ssDNA conjugates, we evaluated the impact of the different conjugation chemistries on the binding between the affinity proteins and their target (HER2) using surface plasmon resonance (SPR) (Fig. 2). To analyse ONT and ONT-Fab, we immobilised HER2 on a dextran-coated gold SPR chip *via* non-selective lysine-carboxylic acid coupling. For the ONT analytes, we utilised a HER2 ligand density corresponding to 150 RU, and analysed the target over a lower concentration range (0.206–16.6 nM); this allowed us to avoid the avidity effects that we observed at higher ligand densities. Due to the stronger binding between ONT and HER2, relatively long dissociation times were employed to ensure an accurate fit of the dissociation rate (Fig. S8†). For the ONT-Fab analytes we employed a HER2 ligand density corresponding to 380 RU, which allowed us to achieve acceptable responses (maximum 80 RU) when analysing the ONT-Fab analytes. In the case of the ADAPT6, we observed unacceptably low binding between the ADAPT6 ligands and the HER2-coated surface prepared *via* direct immobilisation (Fig. S9†). To remedy this, we employed biotinylated HER2 and a streptavidin-coated surface, allowing us to achieve higher HER2 ligand densities (800 RU) and subsequently more appropriate responses (maximum 60 RU) when analysing the ADAPT binders. Since biotinylation of the HER2 was achieved *via* lysine modification, we do not anticipate this would significantly impact the epitope availability of the HER2 on the chip when compared to the direct immobilisation approach. To enable fair comparisons, native, modified, and ssDNA-conjugated ligands for each protein were analysed on the same chip sequentially, under identical conditions.

Due to difficulties in accurately determining the concentration of the ssDNA-conjugated ligands after anion exchange purification, we opted not to purify the constructs prior to SPR analysis. Instead, we determined the concentration of the ligands prior to conjugation, and then adjusted accordingly without additional purification. However, we observed no non-specific binding between the ssDNA and HER2 and confirmed that the presence of ssDNA had no detrimental impact on the specific binding (Fig. S10†). The response curves were globally fit to a 1 : 1 kinetic binding model, and the association (*k*_on_) and dissociation (*k*_off_) rate constants, as well as the observed maximum binding (*R*_maxO_), were computed directly from the fitted curves (Fig. 2b and Table S3†). Notably, at high concentrations of ONT and ONT-Fab analytes we observed minor deviations from the expected fit, possibly due to non-specific interactions between the analytes and the chip surface. However, these deviations are minimal, and do not significantly impact the binding parameters (*k*_on_, *k*_off_), as these were calculated from a global fit of the data. Excluding these curves does not significantly change the data. The extent of mass transport limitations, calculated using mass transport coefficients (*k*_t_) obtained from global fitting of the data to a mass-transport limited model, was negligible in these systems (Table S5†). Thus, following the law of mass action, the dissociation constant (*K*_D_) was computed as the ratio between *k*_off_ and *k*_on_. The theoretical *R*_max_ (*R*_maxT_) was calculated according to eqn (1), accounting for the respective molecular weights of the proteins and the density of HER2 on the chip. These calculations are detailed in Table S4.†1
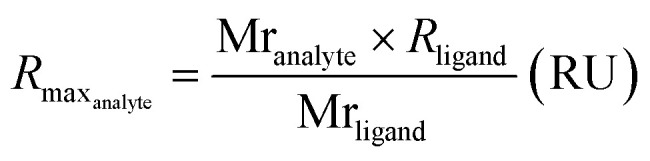


For each protein we studied, both chemical modification and subsequent conjugation to the ssDNA led to a decrease in the apparent association rates (*k*_a_). Interestingly, the fraction of active binders, as estimated from *R*_maxO_ : *R*_maxT_, also decreases upon modification and conjugation, though to a slightly lesser extent. This could be partially responsible for the observed decrease in the association rates, since the calculated association rates depend on the input concentration of active analyte. However, adjusting the concentration of analytes based on the *R*_maxO_ : *R*_maxT_ value does not change the trends significantly. For both *k*_a_ and *R*_maxO_ : *R*_maxT_, the loss was more pronounced when heterogeneous conjugation was employed. In the case of both ONT and ONT-Fab, the dissociation rate (*k*_d_) was largely invariable to any modification. For ADAPT6, modification and conjugation increased the *k*_d_. Once again, this effect was greater when modification occurred *via* lysine residues. It is important to note that all the interactions were studied in PBS (0.1% Tween20) at 25 °C and it is possible that the absolute values for *k*_on_ and *k*_off_ could change under different conditions. However, we would not expect the overall trends to change significantly.

This data suggests that the loss in binding affinity (increase in *K*_D_) observed upon conjugation of the ligands to ssDNA is driven primarily by a decrease in their association with HER2 (*k*_a_). Since the extent of mass transport limitations was negligible in our studies, this cannot be attributed to differences in size between the ligands. Rather, it is more likely a result of steric factors. Given the chemical complexity and bulk of the ssDNA cargos, significant steric interactions between the affinity protein–ssDNA conjugations and HER2 are expected. It is reasonable to assume that these interactions would significantly impact association rates. This hypothesis could also explain why the larger, heterogeneously constructed ONT-Fab–lys–ssDNA_29_, with an affinity protein : ssDNA ratio of approximately 3.5 : 1, had a lower binding affinity than homogeneous ONT-Fab–dis–ssDNA_29_, with a ratio closer to 1 : 1. However, this hypothesis does not explain the relatively lower binding of the heterogeneous ONT–lys–ssDNA_29_ and ADAPT6–lys–ssDNA_29_ conjugates, which both displayed protein : ssDNA ratios that were remarkably similar to their homogeneously constructed counterparts (3.3 : 1 *vs.* 3.2 : 1, and 1 : 1 *vs.* 1.2 : 1, respectively). In these cases, the reduced binding could be a result of modification occurring at, or near, the paratope of the ligand; indeed, both ONT and ADAPT 6 contain lysine residues within their binding interfaces.^40,41^ Conversely, the disulfide- and cysteine-selective chemistries were purposefully chosen so that the ssDNA cargo was positioned far away from the binding site.

These SPR experiments prove that conjugation of ssDNA to each affinity protein significantly influences association kinetics (*k*_on_) and observed maximum binding (*R*_maxO_) between the ligands and HER2. Moreover, these effects are far more pronounced when conjugation occurs non-selectively *via* lysine residues. In each case, faster association rates, lower dissociation equilibrium constants, and higher *R*_maxO_ : *R*_maxT_ ratios are observed compared to non-selective lysine conjugation.

### The impact of DNA length on HER2 binding

**Fig. 3:**
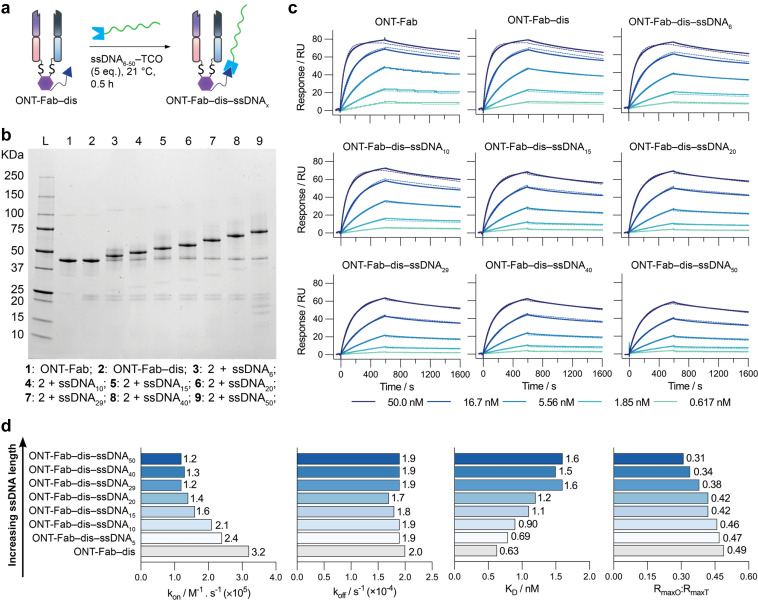
Increasing ssDNA length decreases target binding capacity. (a) A schematic representation of ONT-Fab–ssDNA conjugation. (b) SDS-PAGE analysis of the ONT-Fab–ssDNA_6–50_ conjugates. As the length of the ssDNA increases (left to right), a corresponding increase in the weight of the bioconjugate is observed. (c) SPR sensorgrams for (top left to bottom right) ONT-Fab, ONT-Fab–dis, ONT-Fab–dis–ssDNA_6_, ONT-Fab–dis–ssDNA_10_, ONT-Fab–dis–ssDNA_15_, ONT-Fab–dis–ssDNA_20_, ONT-Fab–dis–ssDNA_29_, ONT-Fab–dis–ssDNA_40_, and ONT-Fab–dis–ssDNA_50_. The association binding kinetics were studied over 600 seconds, and the dissociation kinetics over 1000 seconds. Five concentrations were measured for a single sample of each analyte (solid lines), and each was fit to a 1 : 1 kinetic binding model (dashed lines). (d) Plots of *k*_on_, *k*_off_, *K*_D_, and *R*_maxO_ : *R*_maxT_ for ONT-Fab–dis and ONT-Fab–dis–ssDNA_6–50_. The *k*_on_, *k*_off_, *K*_D_, and *R*_maxO_ values were obtained from a global fit of the data, as described above. *R*_maxT_ was determined using eqn (1). The results are summarised in Table S6.†

After elucidating the impact of conjugation strategy and choice of affinity protein, we next investigated the impact of ssDNA length on the target binding of affinity protein–ssDNA conjugates. We synthesised a selection of ONT-Fab–ssDNA proteins conjugated to ssDNAs of varying lengths (ONT-Fab–dis–ssDNA_6–50_) (Fig. 3a and b) and assessed their binding to HER2 using SPR (Fig. 3c), as described above. The sensorgrams were fit to a 1 : 1 kinetic binding model to determine the binding parameters (*k*_on_, *k*_off_, *K*_D_, *R*_maxO_, *R*_maxT_) (Fig. 3d and Tables S6, S7†).

This data demonstrates that the observed association rates between the ONT-Fab–dis–ssDNA conjugates and HER2 are inversely related to the length of the ssDNA, though dissociation rates remain invariable to ssDNA length. Once again, the extent of mass transport limitation was negligible (Table S8†), suggesting the differences in binding cannot be attributed directly to the size and diffusion of the ligands. Interestingly, the ratio between the observed and theoretical *R*_max_ (*R*_maxO_ : *R*_maxT_) decreases as ssDNA length increases, suggesting a decrease in the fraction of active ligands as a function of ssDNA length. Notably, these effects were not as pronounced as those observed between site-selective and non-selective conjugation (Fig. 2). This data supports the hypothesis that steric factors are a major driver behind the observed decrease in binding affinity; as the size of the DNA increases, so too would any steric effects caused by the DNA. These results suggest that the length of ssDNA cargos should be minimised if maintaining strong target binding in affinity protein–ssDNA conjugates is desirable for an intended application.

### Conjugate performance in immuno-PCR

**Fig. 4:**
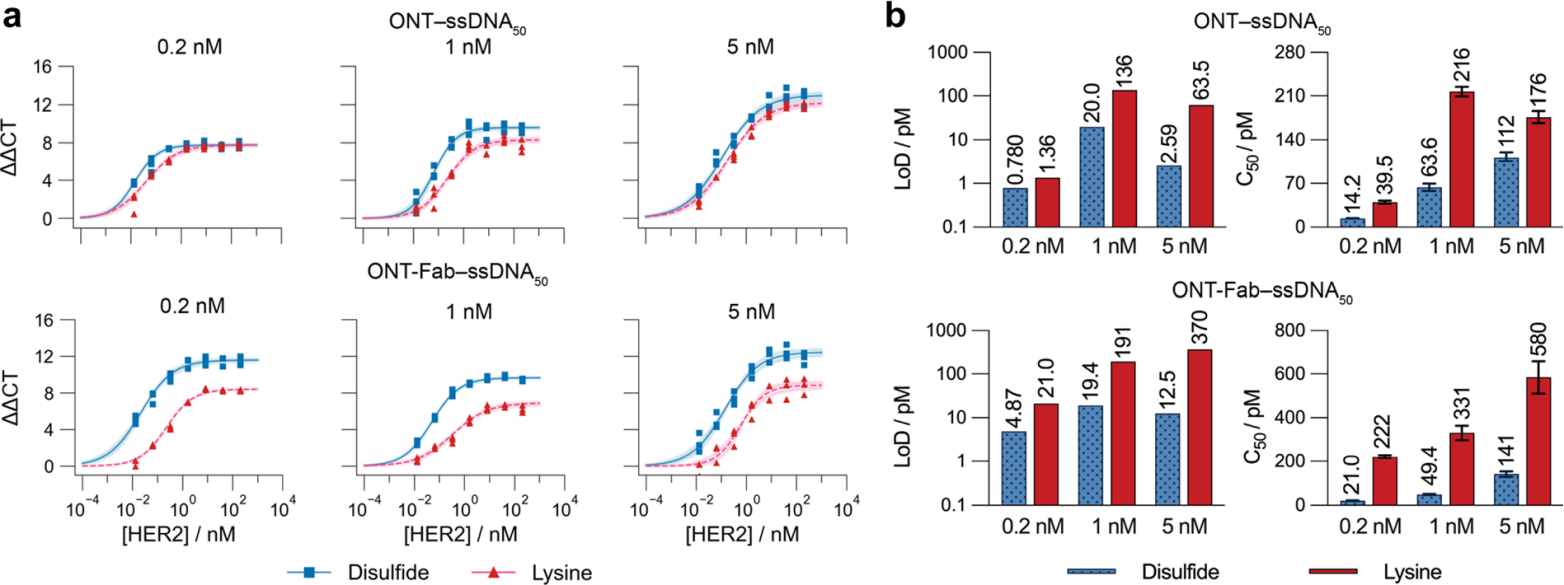
Site-selectively constructed affinity protein–ssDNA conjugates demonstrate improved performance in a model immuno-PCR assay. (a) Standard curves of HER2 titrated against 0.2, 1, or 5 nM of ONT–ssDNA_50_ and ONT-Fab–ssDNA_50_, constructed using either site-selective disulfide chemistry (blue squares) or non-selective lysine conjugation (red triangles). Values are plotted as three individual values, and the shaded regions correspond to the 95% confidence limits of the four-parameter model fit. Raw qPCR curves can be found in Fig. S12.† (b) Plotted values for the limit-of-detection (LoD) ([HER2] = 0 value + three standard deviations) and *C*_50_ for each assay. Values are plotted as the mean of three individual sample measurements, and the error bars correspond to the 95% confidence limits of the four-parameter model fit. In each case, probes constructed using site-selective disulfide chemistry displayed higher specific signals and lower non-specific signals ([HER2 = 0]).

To investigate whether the advantages in target binding achieved through site-selective conjugation of ssDNA to targeting ligands lead to analytical performance benefits, we designed a model sandwich immuno-PCR (*i*PCR) assay to detect HER2. We conjugated an ssDNA target (ssDNA_50_) to ONT and ONT-Fab *via* the optimised site-selective and non-selective strategies to produce the desired affinity protein–ssDNA conjugates. Due to the poor binding between the ADAPT6–lys–ssDNA conjugates and HER2, the ADAPT6 ligand was omitted from further study. SDS-PAGE analysis indicated comparable conversions and protein : ssDNA ratios to those observed during the conjugation of ssDNA_29_ (Fig. S11†). We subsequently employed these affinity protein–ssDNA conjugates as detection probes in the HER2 sandwich immuno-PCR assay. After forming the immunocomplex, we denatured the proteins, detected the released ssDNA_50_ using quantitative PCR (qPCR) (Fig. S12†), and determined the cycle threshold (*C*_t_) values for each HER2 concentration. We plotted the change in *C*_t_ value as a function of HER2 concentration (Fig. 4a) and extracted the *C*_50_ and limit-of-detection (LoD) for each ligand at each concentration (Fig. 4b).

The observed trends agreed with the SPR results and highlight several key differences between the different affinity protein–ssDNA conjugates. Both ONT–ds–ssDNA_50_ and ONT-Fab–ds–ssDNA_50_ display significantly lower *C*_50_ and limit-of-detection (LoD) values than their counterparts constructed using non-specific lysine chemistry. These differences are more pronounced for the ONT-Fab-based ligands, suggesting the conjugation strategy has a larger effect on these proteins. These results are concordant with the *K*_D_ values obtained from the SPR experiments. The high background signals observed with ligands generated using the non-specific conjugation method raises both the limit-of-detection and lower limit-of-quantification of the assays, decreasing the functional range. These experiments demonstrate the advantages of using site-selective conjugation methods to construct affinity protein–ssDNA probes for immuno-PCR assays.

### Conjugate interactions with membrane-bound HER2

**Fig. 5:**
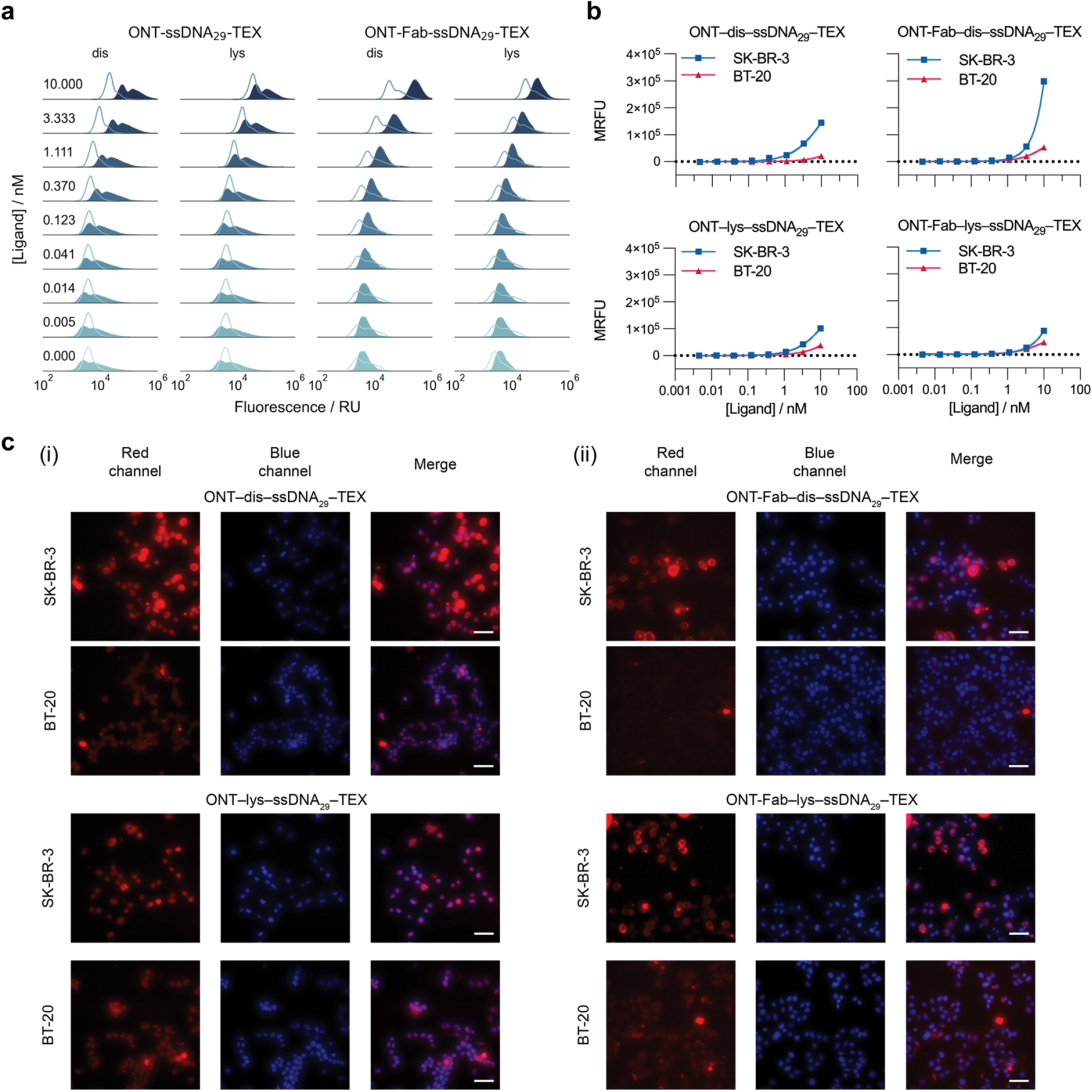
Site-selectively constructed affinity protein–ssDNA conjugates display increased specific and decreased non-specific binding to cell membranes. (a) Normalised fluorescence signal distribution functions of SK-BR-3 (HER2 positive, blue filled) and BT-20 (HER2 negative, clear lines) cells stained with varying concentrations of ONT–dis–ssDNA_29_–TEX, ONT–lys–ssDNA_29_–TEX, ONT-Fab–dis–ssDNA_29_–TEX, and ONT-Fab–lys–ssDNA_29_–TEX. The distributions contain red fluorescence data from live cell populations, which were gated from the dead cells using forward scattering. Distributions comprise 10 000–20 000 measurements from a single population of cells. Contour plots, including details of cell populations and gating, can be found in Fig. S14.† (b) Mean fluorescence value *vs.* ligand concentration for SK-BR-3 (HER2+) and BT-20 (HER2−) cells stained with each ligand. The data was obtained from the corresponding distributions in (a). Error bars (not visible) are plotted as the mean ± SEM. (c) Fluorescence microscopy images showing staining of SK-BR-3 and BT-20 cells using (i) ONT–dis–ssDNA_29_–TEX and ONT–lys–ssDNA_29_–TEX and (ii) ONT-Fab–dis–ssDNA_29_–TEX and ONT-Fab–lys–ssDNA_29_–TEX. Cells were stained with DAPI after fixing. The cells were imaged under 40× magnification using red (E_550_/Em_630_) and blue (Em_377_/Em_442_) filters, a 100 ms exposure, and laser power (SpectraX-6-LCR) at 50%. Scale bar is equal to 50 μm. The data demonstrates that affinity protein–oligonucleotide conjugates constructed using disulfide-selective chemistry have higher specific binding and lower non-specific binding when compared to those constructed using non-specific conjugation.

Previous studies have shown that affinity protein–DNA conjugates suffer from significant non-specific binding to cell membranes.^32^ This can decrease specificity and increase background noise when using such probes to detect membrane-bound proteins. Knowing this, we were interested in investigating how the choice of affinity probe and conjugation approach influences both the specific and non-specific binding of affinity protein–DNA conjugates to their membrane-bound target. To this end, we conjugated ssDNA containing a Texas Red fluorophore (ssDNA_29_–TEX) to ONT and ONT-Fab using both the optimised disulfide- and lysine-directed chemistries (Fig. S13†) and studied their binding interactions with both (SK-BR-3) and (BT-20) cells. SK-BR-3 cells overexpress HER2 (HER2+),^42^ whereas BT-20 cells are triple-negative for breast cancer markers, including HER2 (HER2−).^43^ Thus, these cell lines are ideal models for studying interactions between cells and HER2-targeting ligands. We incubated each probe (4.6 × 10^−3^ to 10 nM) with the cells and then quantified the degree of binding using flow cytometry (Fig. 5a, b and S14†). In the case of the SK-BR-3 cells and ONT ligands, two cell populations were observed. We attribute this second population of cells to a heterogeneous cell staining, whereby certain cells become more strongly stained than others. However, gating the fluorescence to isolate a single population did not affect the observed trend (Fig. S15†). Extending the incubation times alleviated this issue but also led to the death of a significant number of cells. We also studied the interactions between the affinity protein–DNA conjugates and the cells qualitatively using fluorescence microscopy (Fig. 5c). To confirm that differences in binding between the probes and the SK-BR-3 and BT-20 cells could be attributed to the affinity protein–DNA probes, rather than the ssDNA itself, we incubated unconjugated ssDNA_29_–TEX with both cell lines and observed no significant differences, with low total binding (Fig. S16†). As a further control to confirm HER2-mediated binding, we incubated an off-target antibody (anti-EGFR) conjugated to fluorescent ssDNA with both cell populations. Once again, we observed no significant differences between the two cell lines using flow cytometry and fluorescence microscopy (Fig. S17†).

These experiments highlighted several interesting trends. SK-BR-3 cells incubated with ONT–dis–ssDNA_29_–TEX and ONT-Fab–dis–ssDNA_29_–TEX display a higher mean fluorescence value than cells incubated with ONT–lys–ssDNA_29_–TEX and ONT-Fab–lys–ssDNA_29_–TEX. This is despite the fact that ligands constructed using site-specific chemistries exhibited similar fluorescence to those created using non-specific chemistries (Fig. S18†). This suggests that site-specifically constructed probes have a higher affinity for membrane-bound HER2 when compared to probes constructed using non-selective chemistries. We observed the opposite trend with HER2− BT-20 cells; while each of the probes displays some non-specific binding to the cells, particularly at high probe concentrations, the problem was less apparent with probes constructed using site-selective chemistries. Once again, this effect was most pronounced for the ONT-Fab probes. Interestingly, the largest difference in ssDNA : ligand ratio between the site-selective and non-site-selective conjugation strategies (1 : 1 *vs.* 3.5 : 1) was also observed with the ONT-Fab conjugates. This suggests a correlation between the number of ssDNA payloads and the extent of these non-specific interactions. This is unsurprising, as interactions between ssDNA and proteins,^44^ polymeric materials,^45,46^ and cell-surfaces^32^ are well documented. Intriguingly, when analysing the cells using flow cytometry, we observed the highest mean fluorescence with cells labelled with ONT-Fab–dis–ssDNA_29_, despite this probe's relatively low fluorescence and lower binding affinity. This could be attributed to the small size of the Fab fragment, which makes it more amenable to binding to the high density of HER2 receptors on the surface of the SK-BR-3 cells.^47^ The larger size of the IgG may limit binding in such a densely crowded environment, particularly considering the epitope of HER2 is in domain IV, which is held close to the membrane.^34^ These results imply that using site-specific chemistries to furnish affinity protein–DNA conjugates is an effective method for both increasing specific binding to membrane-bound targets and reducing non-specific interactions.

## Conclusions

This work conclusively demonstrates the impact that conjugation to ssDNA has upon the binding between different affinity proteins and their target, the influence of different conjugation strategies upon the magnitude of this impact, and how these factors influence the performance of affinity protein–DNA conjugates as analytical probes. Specifically, our results highlight the detrimental impact that non-specific lysine conjugation has upon the performance of these probes and the benefits of shifting toward site-selective conjugation strategies.

Given the growing interest in developing affinity protein–DNA conjugates and the concurrent rise in the number of analytical assays and biological therapeutics reliant on them, the implications of this work are significant and wide-ranging. The importance of site-selective conjugation on the performance of the Fab–ssDNA_29_ and ADAPT6–ssDNA2_9_ conjugates is particularly interesting. The benefits of using smaller affinity proteins in biosensing/analytical assays are well documented, though they remain relatively underutilised for DNA-driven assays, *e.g.*, iPCR, iRCA and PLA/PEA. Our data suggest that if smaller affinity proteins are to be routinely employed as probes for these assays, it is imperative that they are constructed using site-selective chemistries and that care should be taken in choosing the attachment site and length of the DNA cargo. Overall, the data presented here should serve as a guide for those hoping to design, create, and apply affinity protein–ssDNA conjugates within biosensing and imaging assays.

We hope that this work will inspire similar investigations. Exploring the impact of ssDNA : affinity protein ratio on the binding properties of affinity protein–DNA conjugates would undoubtedly yield exciting results, and many site-selective conjugation strategies exist to facilitate this, *e.g.*, ThioMab,^48^ transglutaminase,^49^ and dual-functionalized dibromopyridazinediones.^50^ A study into the differences between ssDNA and dsDNA payloads would be equally interesting. Moreover, we believe that the combination of disulfide-selective modification and iEDDA “click” chemistry to furnish protein–ssDNA conjugates will pave the way towards a plethora of novel protein–DNA constructs (*e.g.*, bispecifics, protein–siRNA conjugates, protein–DNA origami conjugates).

## Biological samples

All DNA was purchased from Microsynth (Bulgach, Switzerland). Ontruzant was purchased from Samsung Bioepis (Incheon, Republic of Korea). HER2 was purchased from Sinobiological (Beijing, People's Republic of China). Cell lines were originally purchased from ATCC (Manassas, USA).

## Data availability

Experimental protocols and additional data, including raw SDS-PAGE data, can be found in the ESI.†

## Author contributions

Conceptualisation: DAR, ART, FA, JN, P-AN, AJdM; investigation: ART, FA, DAR, JN, AJBH, SDI, IAT, PAS, GS, FMC; data analysis ART, FA, JN, P-AN, DAR, PAS, IAT, SDI, AJBH, GS; writing (original draft): DAR, ART, FA, AJdM, VC; writing (review and editing): DAR, AJdM, ART, FA, PAS, IAT, SDI, VC; data visualisation: DAR, ART; supervision: DAR, AJdM, VC, P-AN; resources: AJdM, DAR, VC. P-AN, NA; funding acquisition: AJdM, DAR, VC, P-AN.

## Conflicts of interest

There are no conflicts to declare.

## Supplementary Material

SC-015-D4SC01838A-s001

SC-015-D4SC01838A-s002
