# Supplementary material for: Site-directed conjugation of single-stranded DNA to affinity proteins: quantifying the importance of conjugation strategy
Source: Chem Sci. 2024 May 7;15(23):8982–92. doi: 10.1039/d4sc01838a (PMC11168188; doi:10.1039/d4sc01838a)
Supplement: SC-015-D4SC01838A-s001 [file SC-015-D4SC01838A-s001.pdf]

Electronic Supplementary Material (ESI) for Chemical Science.
This journal is © The Royal Society of Chemistry 2024

Site-directed Conjugation of Single-Stranded DNA to Affinity Proteins: Quantifying the Importance of Conjugation Strategy

Andres Rocha Tapia,[1‡] Fabrice Abgottspon,[1‡] Johan Nilvebrant[2], Per-Åke Nygren[2], Sarah Duclos Ivetich[1], Andres Javier Bello Hernandez[1], Ioanna A. Thanasi[3], Peter A. Szijj[3], Ghali Sekkat[1], François M. Cuenot[4], Vijay Chudasama[3], Nicola Aceto[4], Andrew J. deMello[1]*, and Daniel A. Richards[1]*

[1]Institute for Chemical and Bioengineering, ETH Zurich, Vladimir-Prelog-Weg 1, 8093 Zürich, Switzerland

[2]Department of Protein Science, KTH Royal Institute of Technology, AlbaNova University Center, 106 91 Stockholm, Sweden

[3]Department of Chemistry, University College London, 20 Gordon Street, WC1H 0AJ London, United Kingdom

[4]Department of Biology, Institute of Molecular Health Sciences, ETH Zurich, Otto-Stern-Weg 7, 8093 Zürich, Switzerland

*Corresponding authors: daniel.richards@chem.ethz.ch, andrew.demello@chem.ethz.ch

‡Authors contributed equally.

Table of Contents

METHODS 2

Buffers 2
Proteins and Antibodies 2
DNA modification 2
Protein–DNA conjugation 3
LC-MS 4
Surface plasmon resonance analysis 5
Immuno-PCR 5
Cell culture 5
Cytometry 6
Preparation of cells for Imaging 6
Cell Imaging Procedure 6

ORGANIC SYNTHESIS & CHEMICAL COMPOUNDS 7

General 7
Schemes 7
NMR spectra 9

PROTEIN MODIFICATION 18

LCMS 18

DNA MODIFICATION 19

SEQUENCES 19
LCMS 20
PROTEIN–SSDNA CONJUGATION 21
OPTIMISATION OF ONT-F(AB)–SSDNA29 CONJUGATION 21
DENSITOMETRY AND UV/VIS SPECTROMETRY ANALYSIS 21
DECREASING THE LYSINE LABELLING OF ONT-FAB 22
SPR ANALYSIS 23
DETERMINING MTL EFFECTS 23
SPR DATA SUMMARY TABLES 25
SUPPORTING SPR DATA 28
IMMUNOPCR 29
SDS-PAGE 29
QPCR 29
CYTOMETRY AND CELL STAINING 30
SDS-PAGE 30
CONTOUR PLOTS 31
ALTERNATIVE GATING 32
CONTROL EXPERIMENTS 33
REFERENCES 35

Methods

Buffers

Borate buffer–EDTA (BB-EDTA) (pH = 8.0, 50 mM borate, 2.0 mM ethylenediaminetetraacetic acid); borate buffer (BB) (pH = 8.4, 50 mm borate); PBST (PBS + 0.1% tween 20); Carbonate buffer (pH = 9.8, 20 – 100 mM carbonate).

Proteins and Antibodies

The exact ADAPT6 used in this study was previously reported as $ADAPT6_{ERBB2=FACS-6}$.[1] The protein was expressed accordingly to the protocol from this original report. Ontruzant (ONT) was produced by Samsung Bioepsis, and provided to UCL Chemistry by University College Hospital. Ontruzant Fab (ONT-Fab) was obtained from ONT according to previously published digestion protocols.[2]

DNA modification

To a solution of azide-modified oligo $ssDNA_x$ (Microsynth, Switzerland) (20 µL, 1 mM in BB-EDTA), TCO–PEG_{12}–DBCO (BroadPharm, USA) (8 equiv., 3.2 µL, 50 mM in DMSO) was added and the solution incubated at 21°C for 1 hour. The DNA was subsequently diluted to a total volume of 30 µL in BB-EDTA and purified using a Micro BioSpin P6 column (Bio-Rad, USA).

Protein–DNA conjugation

Below are general protocols for protein–DNA conjugation. Incubation times and ssDNA lengths and equivalences were changed according to the experiments described above. Ultrafiltration was performed using VivaSpin devices (Sartorius, Germany). UV-Vis spectroscopy or microBCA assay were used to determine protein concentrations, with extinction coefficients; ε_{280} = 215,000 M^{-1} cm^{-1} for ONT, ε_{280} = 68750 M^{-1} cm^{-1} for ONT-Fab, ε_{280} = 7450 M^{-1} cm^{-1} for ADAPT6, and ε_{335} = 9,100 M^{-1} cm^{-1} for pyridazinedione scaffolds. A correction factor at 280 nm of 0.25 (of ε_{335}) was employed to correct for the absorbance of the pyridazinedione scaffold.[3] Appropriate buffers were used as blanks for baseline correction. Affinity protein–ssDNA conjugates were purified using anionic exchange spin chromatography (ThermoFisher Scientific, USA), and eluted into an ammonium acetate buffer (pH = 3.75, 500 mM acetate).

Preparation of ONT–lys–ssDNA

To a solution of Ontruzant (Samsung Bioepis, Republic of Korea) (40 µL, 20 µM, BB pH = 8.4), 6-methyl-tetrazine-PEG5-NHS ester (Jena Biosciences) (10 equiv., 1.60 µL, 5 mM in DMSO) was added and the solution incubated at 37°C for 2 hours. The protein was subsequently purified *via* ultrafiltration (30,000 Da MWCO, 5 × 500 µL) into BB-EDTA to yield ONT–lys. To a solution of this modified antibody (ONT-lys, 20 µL, 11.7 µM), TCO–ssDNA (10 equiv., 9.00 µL, 260 µM in BB–EDTA) was added and the reaction incubated at 21°C for 30 minutes to yield ONT–lys–ssDNA.

Preparation of ONT–dis–ssDNA

To a solution of Ontruzant (50 µL, 20 µM, BB–EDTA), tris(2-carboxyethyl)phosphine hydrochloride (40 equiv., 2.0 µL, 20 mM in BB–EDTA pH = 8.0) was added and the solution incubated at 37°C for 2 hours to yield reduced ONT. The protein was subsequently purified *via* ultrafiltration (30,000 Da MWCO, 5 × 500 µL) into BB–EDTA. To a solution of reduced ONT, dibromopyridazinedione-methyltetrazine (24 equiv., 1.78 µL, 10 mM in DMSO) was added, and the solution was incubated at 21°C for 1.5 hours. The protein was subsequently purified *via* ultrafiltration (30,000 Da MWCO, 6 × 500 µL) into BB-EDTA to yield ONT–dis. To a solution of ONT-dis (15 µL, 17.8 µM in BB-EDTA), TCO–ssDNA (10.0 equiv., 10.3 µL, 260 µM in BB–EDTA) was added and the reaction was incubated at 21°C for 30 minutes to yield ONT–lys–ssDNA.

Preparation of ONT-Fab–lys–ssDNA

To a solution of Ontruzant Fab (20 µL, 40 µM, BB pH = 8.4), 6-methyl-tetrazine-PEG5-NHS ester (10 equiv., 0.8 µL, 10 mM in DMSO) was added and the solution was incubated at 21°C for 2 hours to yield ONT-Fab–lys. The sample was diluted to a total of 120 uL in BB–EDTA and purified using a ZebaSpin 7 kDa MWCO column (Thermo Fisher Scientific, USA). The protein was subsequently purified a second time *via* ultrafiltration (30,000 Da MWCO, 2 × 500 µL) into BB-EDTA. To a solution of ONT-Fab-lys (13 µL, 15.0 µM in BB-EDTA), TCO–ssDNA (6.0 equiv., 4.50 µL, 260 µM in BB–EDTA) was added and the reaction was incubated at 21 °C for 30 minutes to yield ONT-Fab–lys–ssDNA.

Preparation of ONT-Fab–dis–ssDNA

To a solution of Ontruzant Fab (80 µL, 20 µM, BB–EDTA), tris(2-carboxyethyl)phosphine hydrochloride (10 equiv., 1.6 µL, 20 mM in BB–EDTA pH = 8.0) was added and the solution incubated at 37°C for 2 hours to yield reduced ONT-Fab. The protein was then purified *via* ultrafiltration (30,000 Da MWCO, 5 × 500 µL) into BB–EDTA. The sample was purified again using a ZebaSpin 7 kDa MWCO column. To a solution of reduced ONT-Fab (59 µL, 20.0 µM in BB-EDTA), dibromopyridazinedione-methyltetrazine (8 equiv., 3.78 µL, 2.5 mM in DMSO) was added and the solution incubated at 21°C for 1.5 hours to yield ONT-Fab–dis. The protein was diluted to 80 uL into BBS pH=8.0 and purified using a ZebaSpin 7 kDa MWCO column. To

a solution of ONT-Fab-dis (16.0 µL, 16.0 µM in BB-EDTA), TCO–ssDNA (6.0 equiv., 5.9 µL, 260 µM in BB–EDTA) was added and the reaction was incubated at 21°C for 30 minutes to yield ONT-Fab–dis–ssDNA.

Preparation of ADAPT6–lys–ssDNA

To a solution of ADAPT6 (20 µL, 205 µM, BB pH = 8.4), 6-methyl-tetrazine-PEG5-NHS ester (10 equiv., 0.82 µL, 50 mM in DMSO) was added and the solution incubated at 21°C for 2 hours to yield ADAPT6–lys. The sample was purified via ultrafiltration (3000 MWCO, 4 × 500 µL) into BB-EDTA. To a solution of ADAPT6–lys (10 µL, 32.5 µM in BB-EDTA), TCO–ssDNA (6.0 equiv., 7.5 µL, 260 µM in BB–EDTA) was added and the reaction was incubated at 21°C for 30 minutes to yield ADAPT6–lys–ssDNA.

Preparation of ADAPT6–cys–ssDNA

To a solution of ADAPT6 (20 µLµ, 205 µM, BB-ED–EDTA pH = 8.0), 6-methyl tetrazine-PEG4-maleimide (10 equiv., 0.82 µL, 50 mM in DMSO) was added and the solution incubated at 21°C for 1.5 hours to yield ADAPT6–cys. The sample was purified via ultrafiltration (3000 MWCO, 4 × 500 µL) into BB-EDTA. To a solution of ADAPT6–cys (10 µL, 32.5 µM in BB-EDTA), TCO–ssDNA (6.0 equiv., 7.5 µL, 260 µM in BB–EDTA) was added and the reaction was incubated at 21°C for 30 minutes to yield ADAPT6–ds–ssDNA.

SDS-PAGE

SDS-PAGE was performed on 4 – 20% SDS-PAGE gels (Bio-Rad, USA). Samples (IgG 2.5 mM, Fab 5 mM, ADAPT6 44 mM in borate buffer-EDTA) were pre-mixed 4:1 with 4× Laemmli sample buffer (Bio-Rad, USA), incubated at 95°C for 5 minutes, and then centrifuged (10,000 r.c.f, 2 minutes). The samples (5 µL) were loaded onto the stacking gel, and electrophoresis was performed (100 V for 10 minutes, 200 V for 30 minutes). A PageRuler™ Protein Plus ladder (Thermo Fisher Scientific, USA) was included. The gel was visualised using the stain-free protocol on a GelDoc Go (Bio-Rad, USA) or stained using QuickBlue (Lubio Science, Switzerland). Images of QuickBlue stained gels were obtained on a GelDoc Go.

LC-MS

Protein LC-MS

LC-MS was performed on protein samples using a Waters Acquity uPLC connected to Waters Xevo G2 QTof. Column: Acquity UPLC® Protein BEH C4, 1.7 µm, 2.1 × 50 mm. The column temperature was held at 40°C. Wavelength: 210–300 nm. Mobile Phase: Water (0.1% formic acid):MeCN (0.1% formic acid); 95:5 gradient over 6 minutes to 5:95. Flow Rate: 0.4 mL/min. Injection volume 5 µL. MS Mode: ES+. Scan Range: m/z = 300–4000. Scan time: 0.5 seconds. The electrospray source of the MS was operated with a capillary voltage of 3.0 kV and a cone voltage of 25 V. Nitrogen was used as the nebuliser and desolvation gas at a total flow of 1000 L/h.

Oligonucleotide LC-MS

LC-MS was performed on protein samples using a Waters Acquity uPLC connected to Waters Xevo G2 QTof. Column: Acquity™ Premier Oligonucleotide BEH C18, 1.7 µm, 2.1 × 100 mm. The column temperature was held at 60°C. Wavelength: 210–400 nm. Mobile Phase: Water (4.1% HFIP, 0.7% TEA):MeOH; 100:0 gradient over 7 minutes to 50:50. Flow Rate: 0.5 mL/min. Injection volume 5 µL. MS Mode: ES-. Scan Range: m/z = 500–4000. Scan time: 0.5 seconds. The electrospray source of the MS was operated with a capillary voltage of 0.71 kV and a cone voltage of 40 V. Nitrogen was used as the nebuliser and desolvation gas at a total flow of 300 L/h.

Surface plasmon resonance analysis

Surface plasmon resonance experiments were run on Biacore 3000 and T200 instruments (GE Healthcare) at 25 °C with PBST as a running buffer. HER2 (SinoBioligical, China) was diluted to 10 μg ml^{-1} in 10 mM NaOAc pH 4.5 and immobilised on CM5 chips by amine coupling. Alternatively, biotinylated HER2 (SinoBiological, China) was diluted to 3 μg/mL and immobilised onto a streptavidin-coated CM5 chip. Immobilisation levels are detailed in Tables S4 and S7. The analytes were diluted into PBST and injected at 30 μl min^{-1}. Surfaces were regenerated using 10 mM HCl. Sensorgrams were double-referenced using a blank flow cell and a buffer injection. Data were fitted to a Langmuir 1:1 interaction using BiaEval 4.1 software, and dissociation equilibrium constants were calculated from the association and dissociation rate constants. As a negative control to rule out non-specific binding, TCO–ssDNA$_{29}$ was diluted to 400 nM in PBST and injected (no response observed).

Immuno-PCR

Immuno plate-based assay

A sandwich-style immuno-assay was performed on 384-well NUNC Maxisorp flat-bottomed black plates (Thermo-Fisher Scientific) in triplicate. Capture antibody (Pertuzumab Biosimilar) (Proteogenix) was added to each well (50 μL, 2 μg / mL in carbonate buffer), a coverslip was placed over the wells, and the plate was incubated at 4°C overnight. The wells were washed four times (100 μL, PBST). HER2 was added as a dilution series to each well (40 μL, 0.0128–200 nM in PBST 1% BSA) and incubated at 21°C for 1 hour. As a control, one well was filled with PBST 1% BSA. The wells were then washed four times (100 μL, PBST). Affinity protein-ssDNA conjugate was added to all wells (40 μL, 0.2–5 nM in 0.5X PBST 0.1% BSA) and incubated at 21°C for 30 minutes. The wells were finally washed six times (100 μL, PBST). Collection solution was added to all wells (50 μL, 0.5X PBST 0.1% BSA) and incubated at 95°C for 20 minutes. Samples were collected and stored for PCR analysis on collection tubes blocked with 0.5X PBST 0.1% BSA (100 μL, 30-minute incubation).

qPCR

qPCR was performed on the samples from the sandwich assay using a QuantStudio 3 Real-Time PCR system (Thermo Fisher Scientific, USA). A PCR MasterMix was prepared using DreamTaq Hot Start PCR Master Mix (Thermo Fisher Scientific, USA), containing 1X EvaGreen (Biotium, USA), 50 nM ROX reference dye (Biotium, USA), and 1 nM forward/reverse primers (Microsynth, Switzerland). The samples obtained from the plate-based sandwich assay were diluted 1 in 2 with UltraPure DNA/RNAse-free distilled water (Thermo-Fisher Scientific). The diluted samples were added to the prepared PCR mix (10 μL sample, 15 μL MasterMix), and qPCR was performed under the following cycling conditions: 95°C for 30 seconds; 40 cycles of 95°C for 7 seconds, 67°C for 30 seconds, 72°C for 10 seconds; final extension step for 10 seconds. The fluorescence data were processed using a Python script developed using Pycharm Professional Edition software (JetBrains, Czech Republic) employing Matplolib, Numpy, Pandas, Seaborn and Scipy packages.

Cell culture

Breast cancer cell lines were provided by François M. Cuenot (Aceto lab, ETHZ), and originally purchased from ATCC. Cell lines tested negative for mycoplasma contamination before commencement of the cell studies (March 2023). The SK-BR-3 cell line (ATCC HTB-30, 43-year old white human female) was cultured in RPMI 1640 (Roswell Park Memorial Institute, Thermo Fischer Scientific, USA), while the BT-20 (ATCC HTB-19, 74-year old white human female) cell line was cultured in Dulbecco's Modified Eagle Medium: Nutrient Mixture F12 (Thermo Fischer Scientific, USA). Both culture media received a 10% supplementation of fetal bovine serum (FBS) and 5% penicillin-streptomycin (Invitrogen, USA). They were maintained during

culture at 37°C in an incubator (Galaxy 170 S, New Brunswick Scientific, USA) with an atmosphere of 95% air and 5% CO_2. Cells were cultured until they reached approximately 90% confluency, as determined by visual observation, after which they were harvested using a 0.25% trypsin-EDTA mix (Thermo Fischer Scientific, USA). Once harvested, the cells were washed with their medium at 160 r.c.f for 5 minutes. The supernatant was removed, and the cells were resuspended in medium to continue the culture or in FACS buffer for further experiment.

Cytometry

Cultured cells were transferred (concentration = 5 x 10^4) to a V-bottom 96-well plate (Cellstar®, Greiner Bio-One, Austria). Cells were washed once with FACS buffer after centrifugation at 160 r.c.f for 5 minutes. The supernatant was removed, and the cells were resuspended with the affinity protein–$ssDNA_{29}$–TEX conjugate or control $ssDNA_{29}$–TEX solutions (100 µL, 0.0046–10 nM, in FACS buffer) and incubated in the dark at 21°C for 30 minutes. As a control, the cells were also incubated with pure FACS buffer. The cells well were washed with FACS buffer to remove unbound protein. The cells were subsequently analysed using a CytoFLEX S (Beckman Coulter, USA). Each sample was measured at a 30 µL/min flow rate and detected based on their forward scattering height (FSH) (factory default values). The data were subsequently analysed with FlowJo Software v10 (BD Life Sciences, USA) and a Python script developed using Pycharm Professional Edition with Matplotlib, Numpy, Pandas and Seaborn packages.

Preparation of cells for Imaging

Cultured cells were seeded into a glass-bottom 18 well µ-Slide (ibidi GmbH, Germany) at a concentration of 10^4 cells per well, with each well containing their respective growth media (100 µL). Following a 24-hour incubation period, the cells were gently washed (PBS, 2 × 100 µL) to remove any non-adherent or excess media. Subsequently, the cells were blocked (1% BSA 0.1% Sperm Salmon DNA in PBS, 100 µL) and incubated at room temperature for 60 minutes under gentle shaking. The cells were washed (PBS, 2 ×100 µL), and each cell group was stained with the protein-conjugate solution (50 nM in PBS) and incubated at room temperature for 80 minutes under gentle shaking. As a control, a group of cells underwent a similar incubation with PBS alone. After incubation, the cells were washed (PBS, 3 × 100 µL). Paraformaldehyde (100 µL, 4% in PBS, Bio-Rad) was added to each well and incubated for 5 minutes. The cells were then washed (PBS, 1 × 100 µL, 2 min incubation). Subsequently, the cell membrane was permeated with Triton-X (100 µL, 0.1% in PBS) and incubated for 5 minutes before the cells were washed (PBS, 3 × 100µL, 2 min incubation). Following that, the nuclei were stained with DAPI (100 µL, 1 µg/mL in PBS) and incubated for 5 minutes before being washed (PBS, 2 × 100 µL). As a control, a group of cells underwent a similar incubation with PBS alone. Finally, to prevent cell desiccation, PBS was added to each well (100 µL).

Cell Imaging Procedure

The cells were imaged on a Ti Eclipse fluorescence microscope (Nikon, Japan) equipped with a SpectraX-6-LCR light source (Lumencor, USA) and a Nikon S Plan Fluor ELWD 40x/0.60 Objective (Nikon, Japan). To visualise Texas Red fluorescence, a filter cube setup with a 550/49 excitation filter, a 630/69 emission filter and a FF593-Di03 dichroic mirror was used. To visualise DAPI fluorescence, a filter cube setup with a 377/50 excitation filter, a 442 long pass emission filter and a FF409-Di03 dichroic mirror was used. To visualise FAM fluorescence, a filter cube setup with a 478/28 emission filter, a 525/45 excitation filter and a HC500 dichroic mirror was used. Images were captured by a C11440 ORCA-Flash 4.0 fluorescence camera (2048x2048 pixels, Hamamatsu Photonics, Japan) with a 100 millisecond exposure time and light power at 50%. Micro-manager software (v1.4.22, Arthur Edelstein *et al.* [4]) was used to control the camera and light source simultaneously. The images were processed using the Fiji software (v2.14.0, Schindelin *et al.*[5]).

Organic Synthesis & Chemical Compounds

General

Unless stated otherwise, all reagents and starting materials were obtained from chemical suppliers and were used as received. Reactions were monitored by thin layer chromatography using pre-coated SIL G/UV 254 plates purchased from VWR. Flash chromatography was carried out manually using Kieselgel 60 M 0.04/0.063 mm silica gel or automatically using a BioTage Isolera with KP-Snap or KP-Sil columns. NMR spectra were recorded using a Bruker AC300, AC500, or AC600 spectrometer (300 MHz, 500 MHz, and 600 MHz, respectively). Chemical shifts (δ) are given in ppm units relative to the solvent reference and coupling constants (J) are measured in Hertz. Proton (1H) NMR multiplicities are shown as s (singlet), d (doublet), t (triplet), q (quartet), m (multiplet), dd (double doublet), dt (double triplet), etc. HMBC, HSQC, and DEPT were employed to aid with accurate assignments.

Schemes

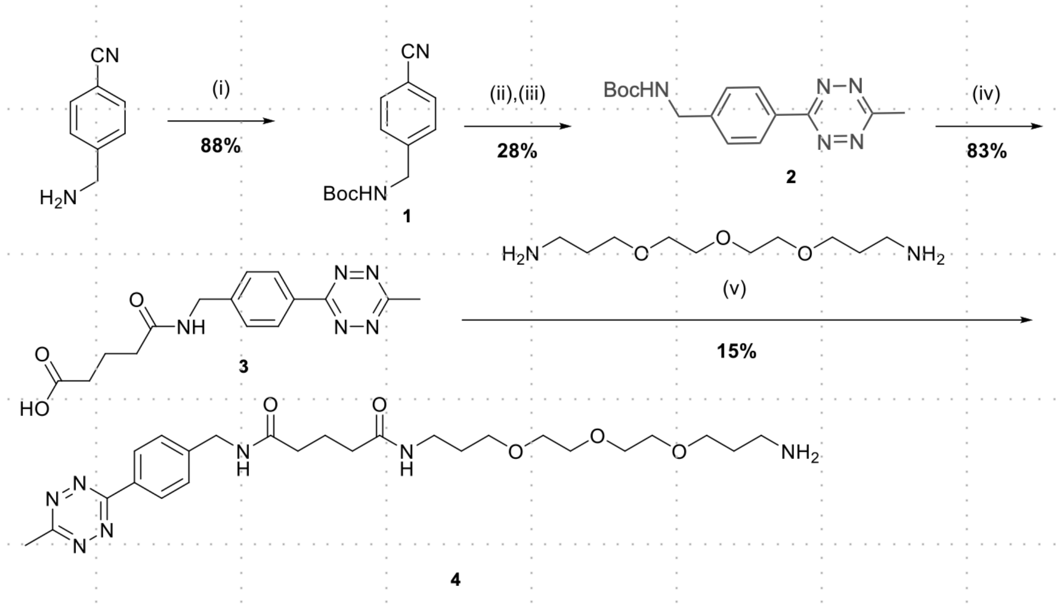

Fig. S1a. Synthesis of methyltetrazine–OEG–amine **4**. *Reagents and conditions*: (i) Di-*tert*-butyl dicarbonate, NaOH, H_2O, 21 ºC, 16 h. (ii) $Zn(OTf)_2$, 1,4-dioxane, MeCN, 65 ºC, 72 h. (iii) $NaNO_2$, AcOH, DCM, 21 ºC, 15 min. (iv) Glutaric anhydride, THF, 55 ºC, 16 h. (v) 3-{2-[2-(3-aminopropoxy)ethoxy]ethoxy}propan-1-amine, NEt_3, HATU, DCM, 21 ºC, 16 h.

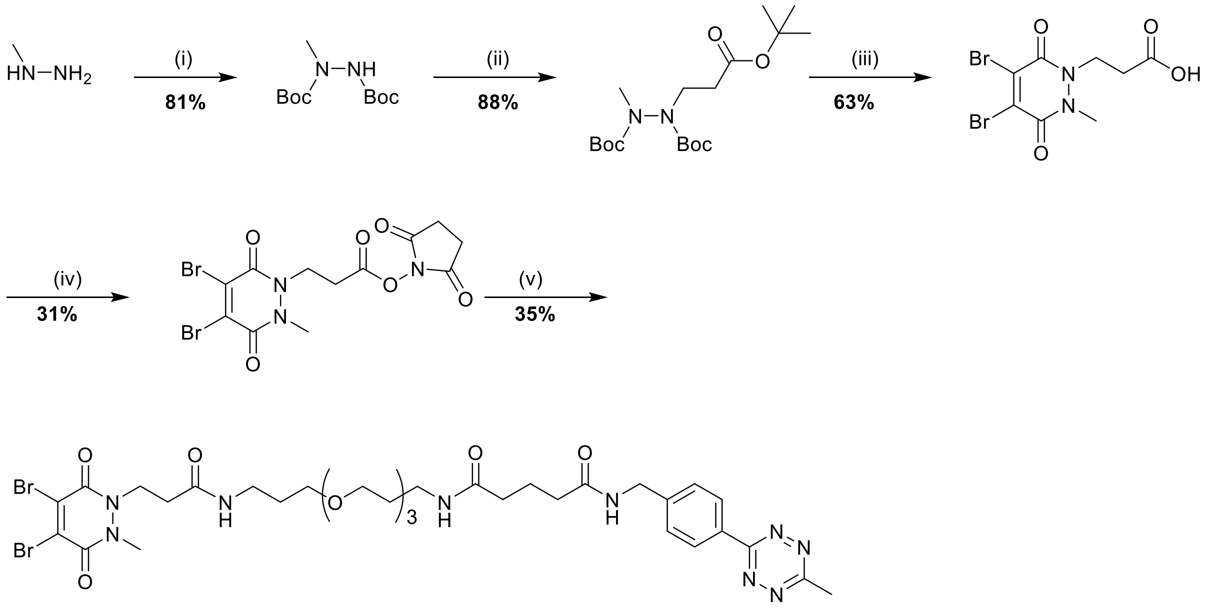

Fig S1b. Synthesis of methyltetrazine–Br_2PD.[6] *Reagents and conditions*: (i) Di-*tert*-butyl decarbonate, propan-2-ol, DCM, 21 ºC, 16 h. (ii) *tert*-butanol, 10% $NaOH_{(aq.)}$, *tert*-butyl acrylate, 60 ºC, 24 h. (iii) Dibromomaleic anhydride, AcOH, reflux, 4 h. (iv) DCC, NHS, dry THF, 21 ºC, 16 h. (v) methyltetrazine–OEG–amine, NEt_3, DCM, 21 ºC, 3 h.

NMR spectra

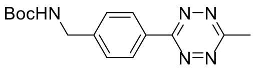

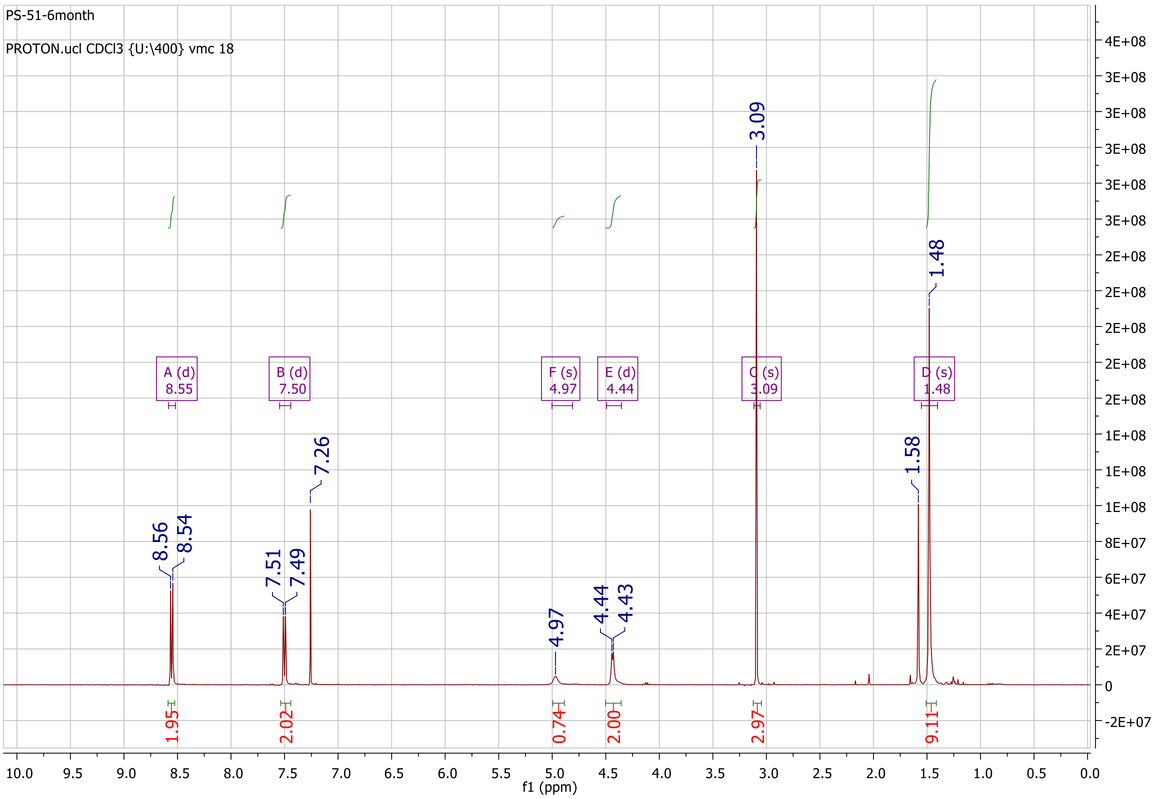

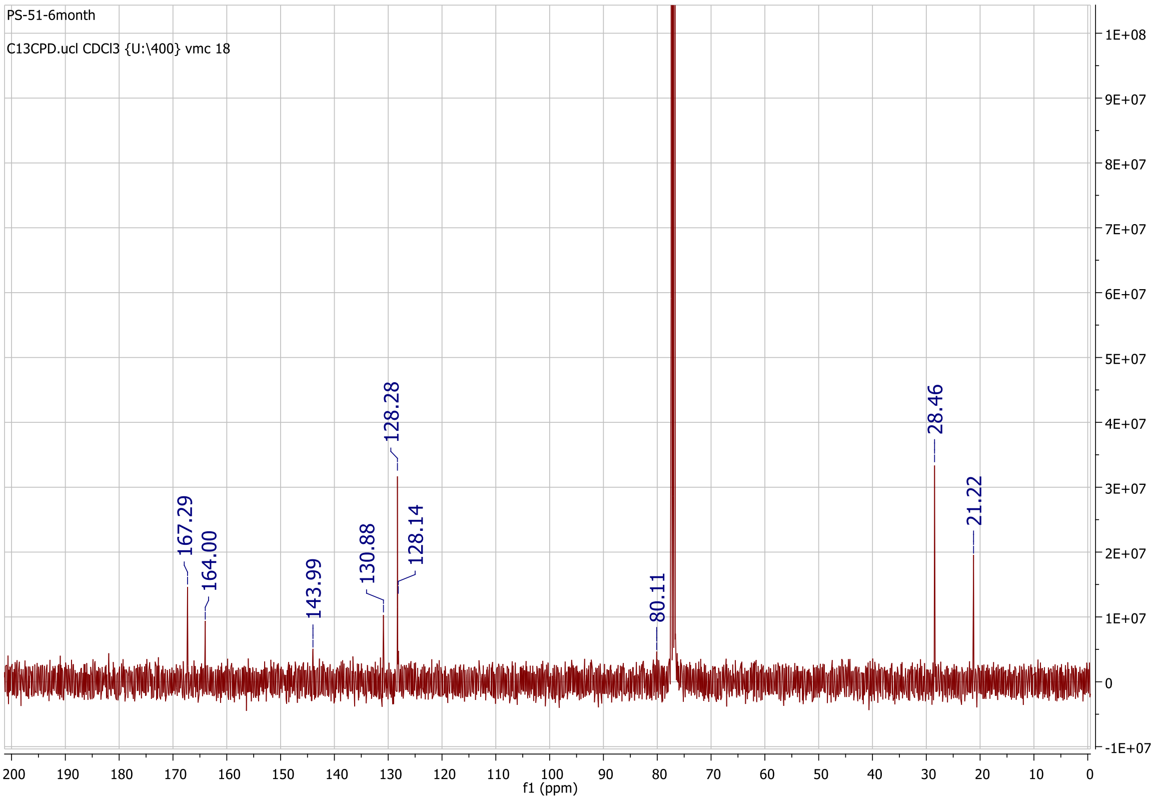

Fig. S1c. ^{1}H and ^{13}C spectra for *tert*-butyl (4-(6-methyl-1,2,4,5-tetrazin-3-yl)benzyl)carbamate.

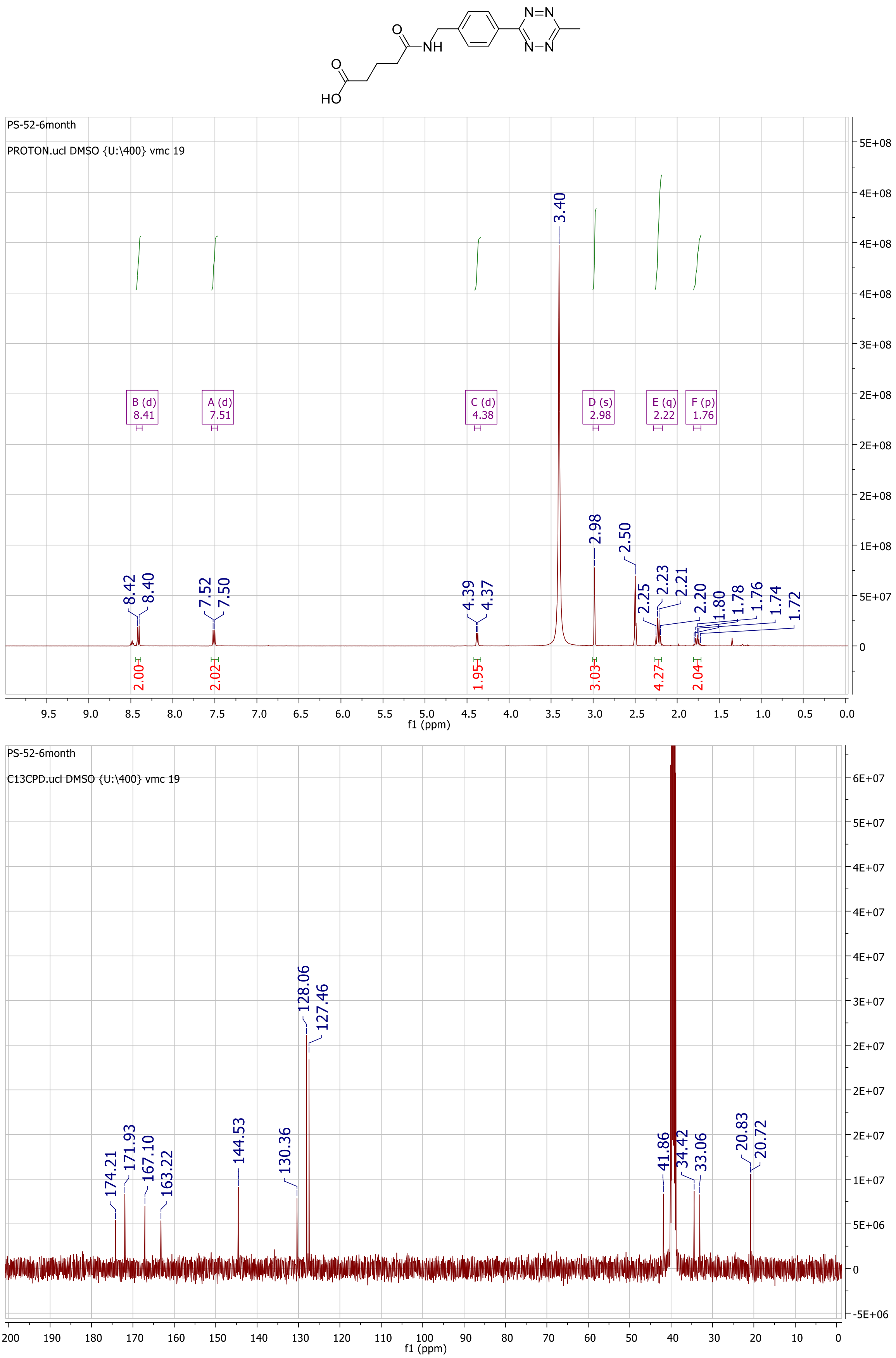

Fig. S1d. ^{1}H and ^{13}C spectra for 5-((4-(6-methyl-1,2,4,5-tetrazin-3-yl)benzyl)amino)-5-oxopentanoic acid.

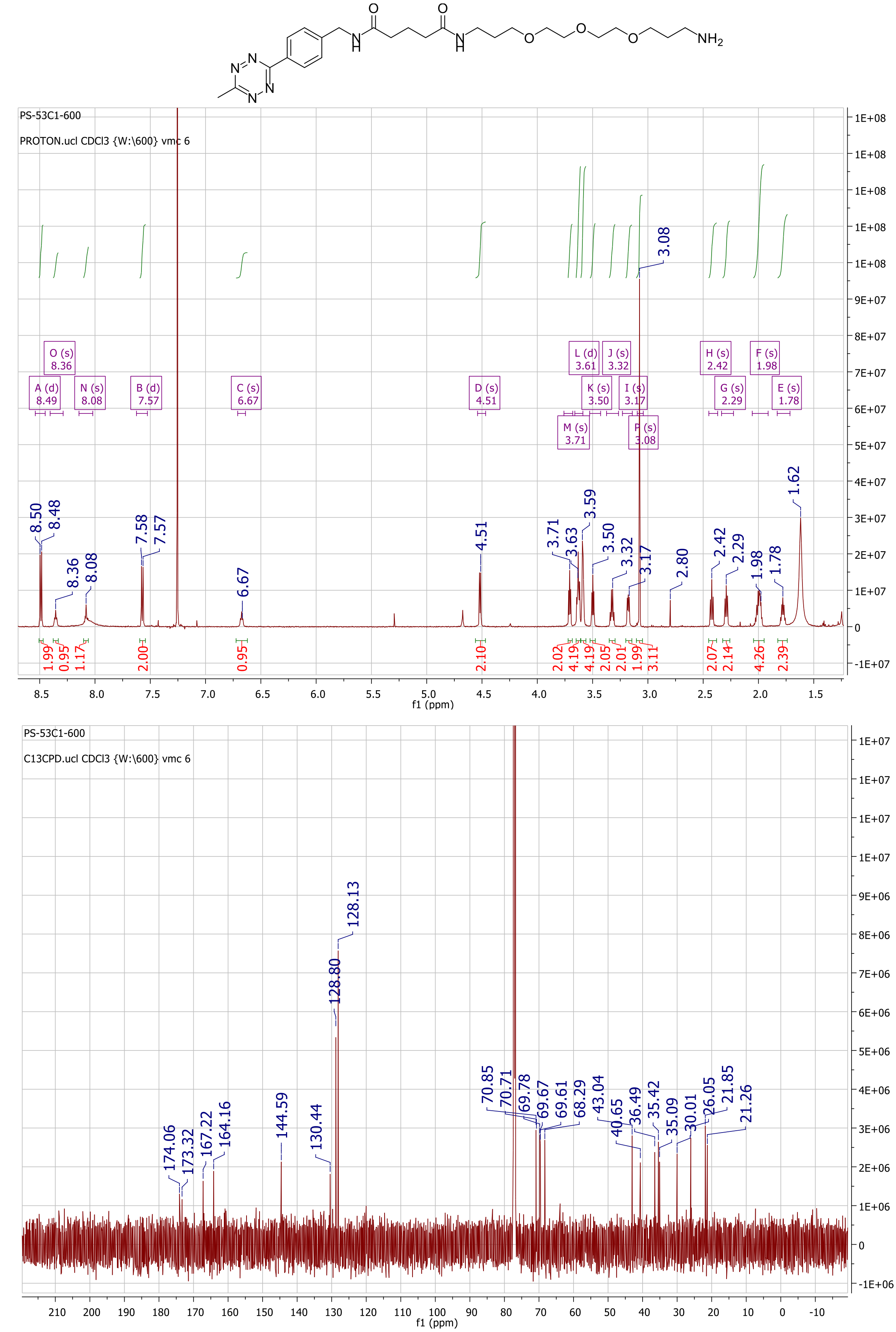

Fig. S1e. ^{1}H and ^{13}C spectra for N^{1}-(3-(2-(2-(3-aminopropoxy)ethoxy)ethoxy)propyl)-N^{5}-(4-(6-methyl-1,2,4,5-tetrazin-3-yl)benzyl)glutaramide.

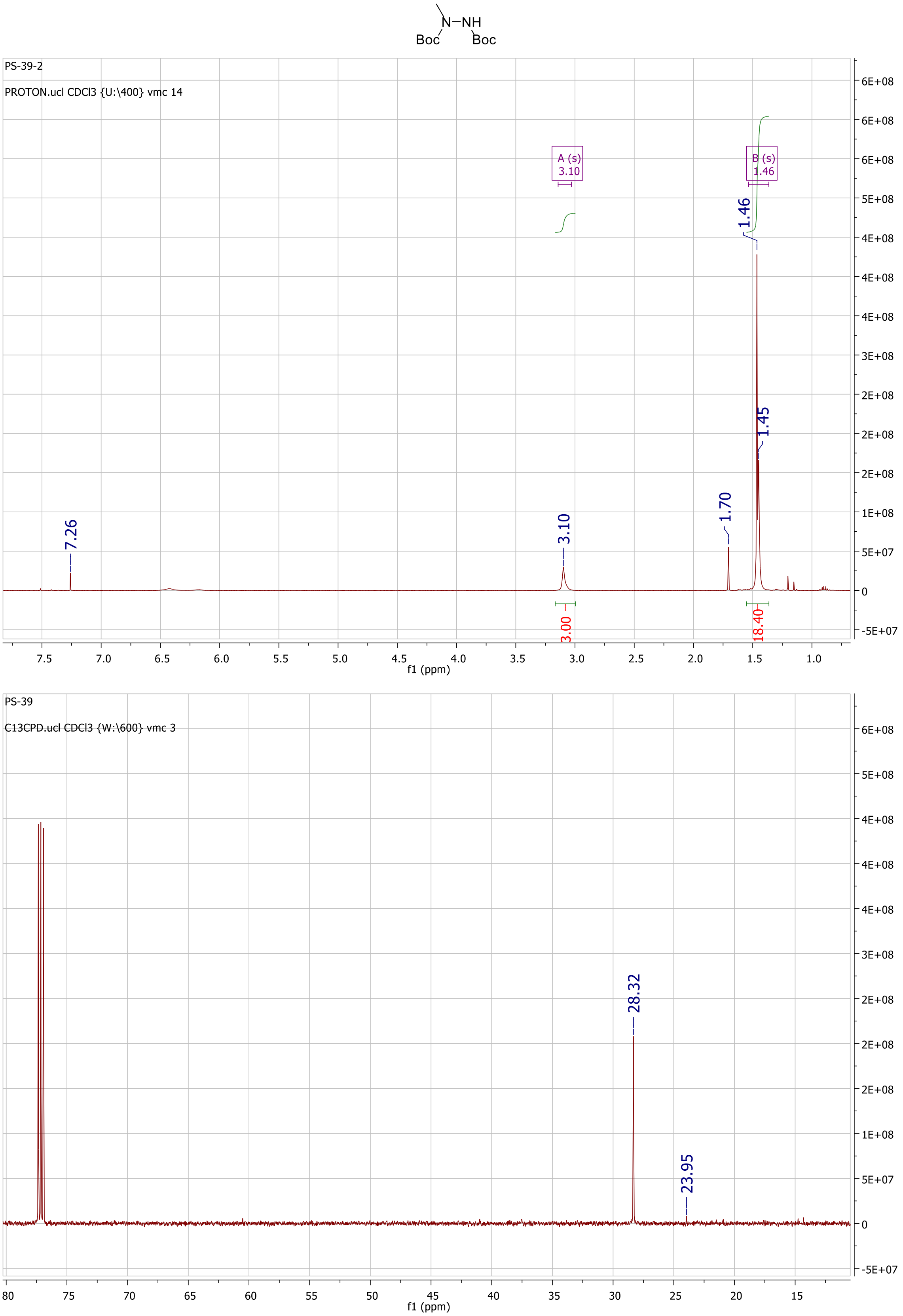

Fig. S1f. ^{1}H and ^{13}C spectra for di-*tert*-butyl 1-methylhydrazine-1,2-dicarboxylate.

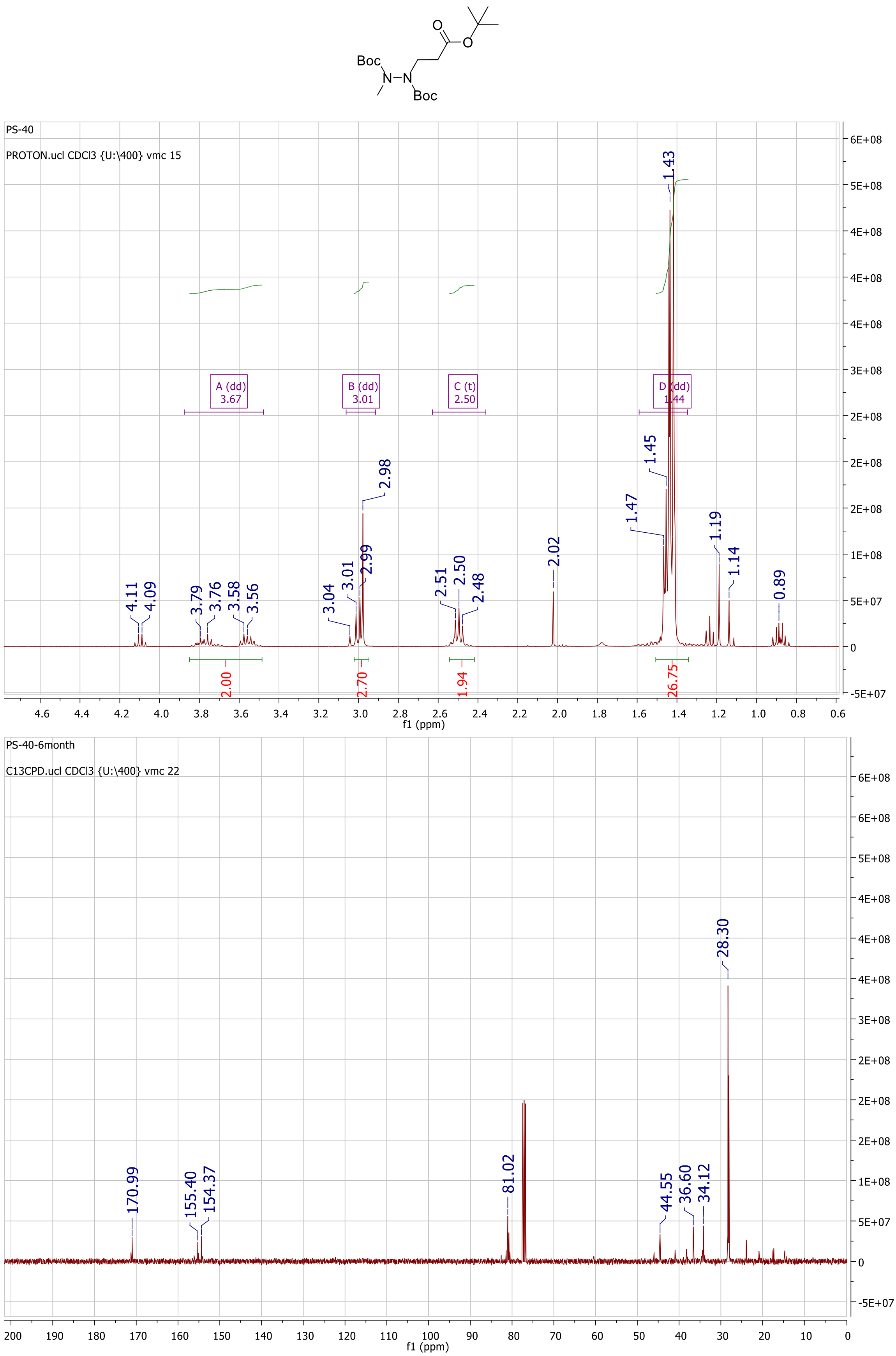

Fig. S1g. ^{1}H and ^{13}C spectra for di-*tert*-butyl 1-(3-(*tert*-butoxy)-3-oxopropyl)-2-methylhydrazine-1,2-dicarboxylate**.**

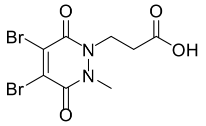

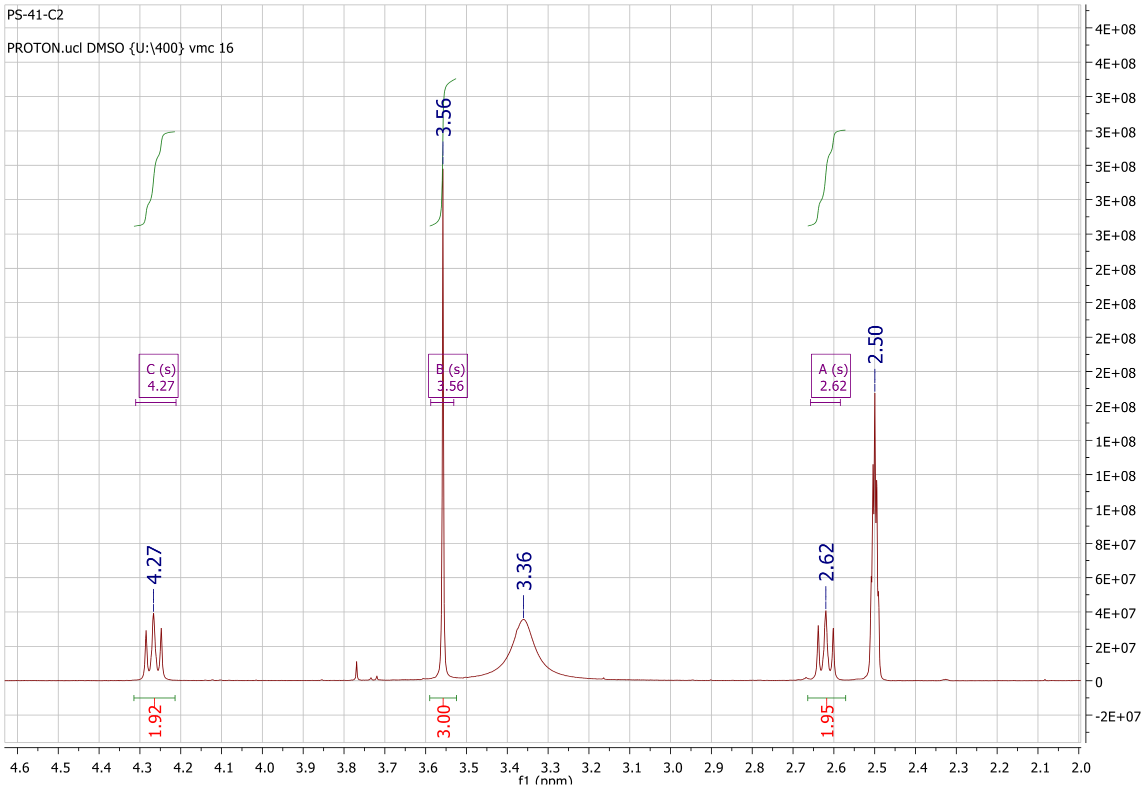

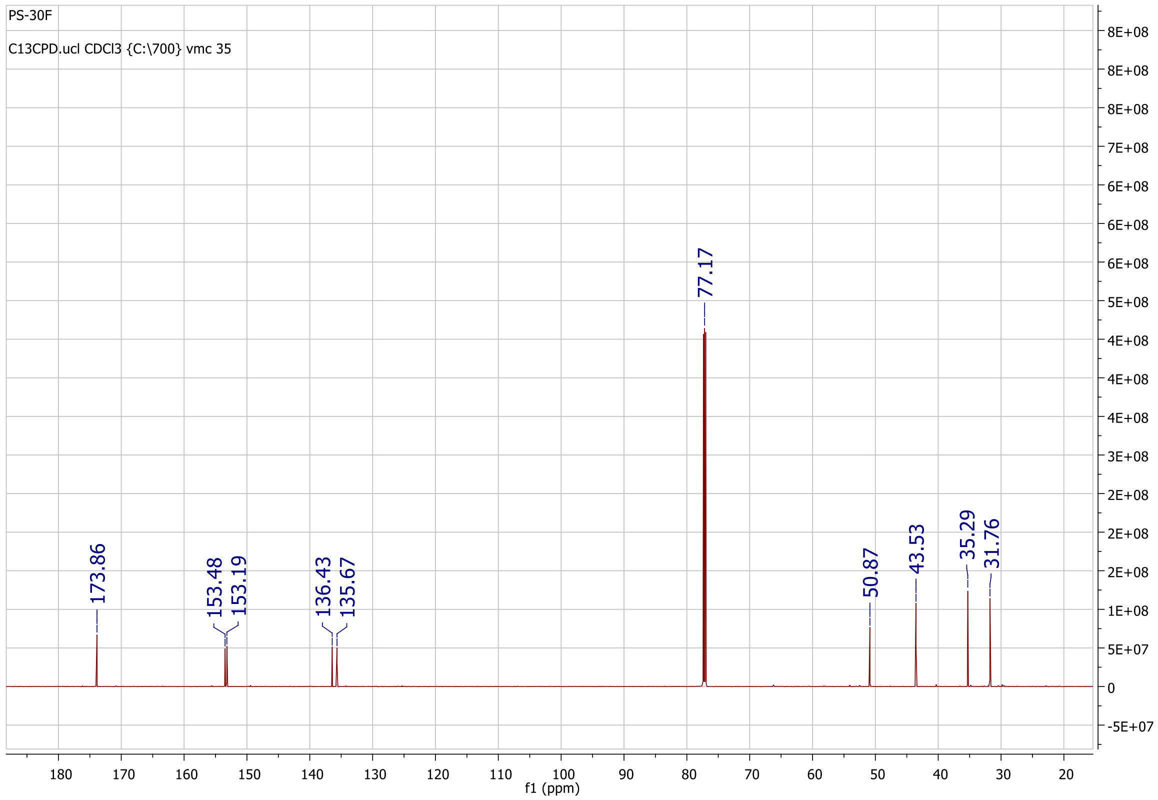

Fig. S1h. ^{1}H and ^{13}C spectra for 3-(4,5-dibromo-2-methyl-3,6-dioxo-3,6-dihydropyridazin-1*(2H)-yl*)propanoic acid.

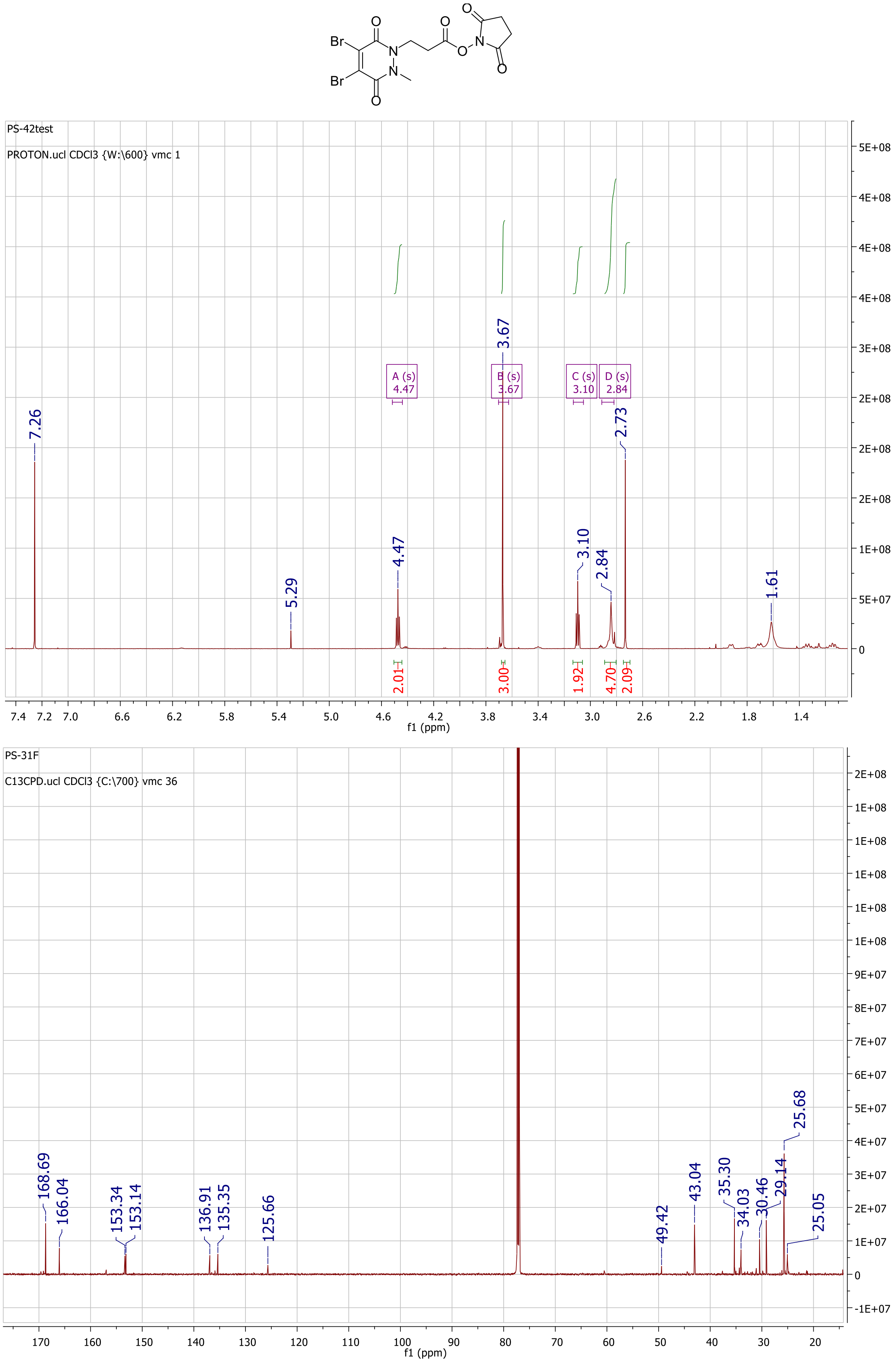

Fig. S1i. ^{1}H and ^{13}C spectra for 2,5-dioxopyrrolidin-1-yl 3-(4,5-dibromo-2-methyl-3,6-dioxo-3,6-dihydropyridazin-1*(2H)-yl*)propanoate.

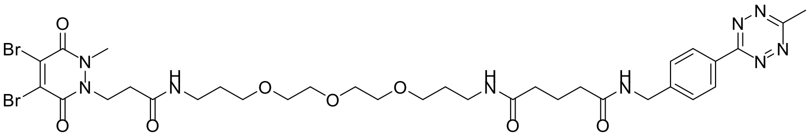

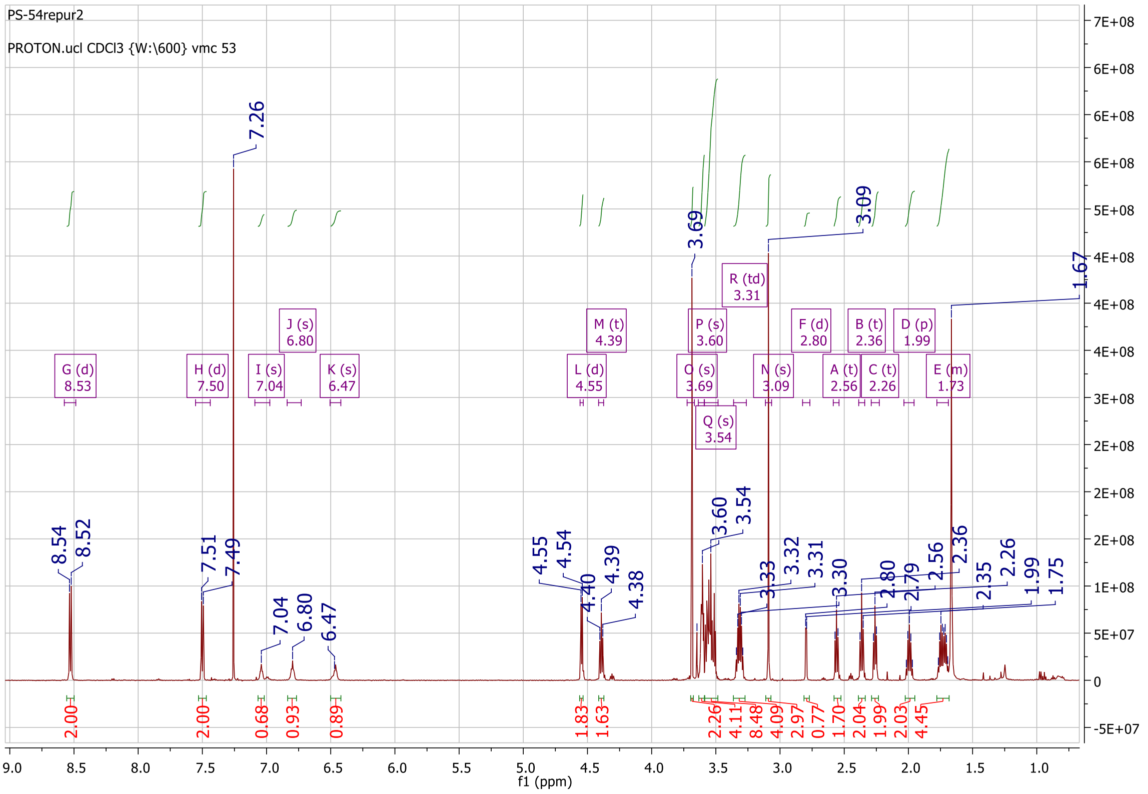

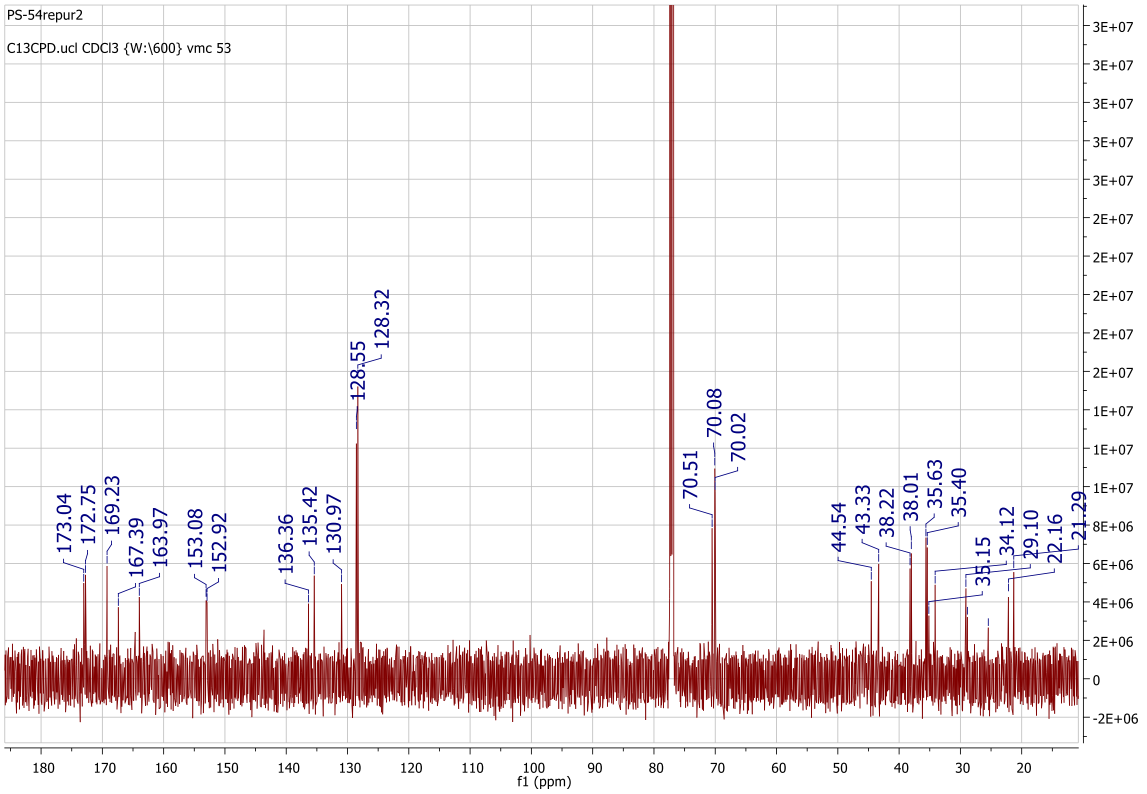

Fig. S1j. ^{1}H and ^{13}C spectra for N^{1}-(17-(4,5-dibromo-2-methyl-3,6-dioxo-3,6-dihydropyridazin-1*(2H)-yl*)-15-oxo-4,7,10-trioxa-14-azaheptadecyl)-N^{5}-(4-(6-methyl-1,2,4,5-tetrazin-3-yl)benzyl)glutaramide.

Table S1. Names, structures, and molecular weights for the chemical linkers used in this study.

Name	Structure	Theoretical molecular mass (g × mol^{-1})
Br_2PD-methyltetrazine	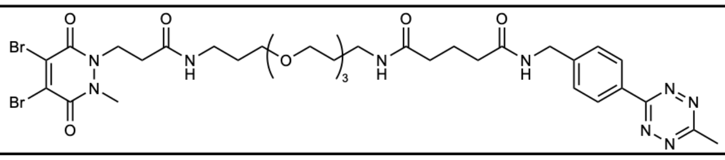	857.6
NHS-methyltetrazine	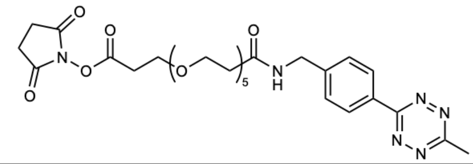	620.3
Maleimide-methyltetrazine	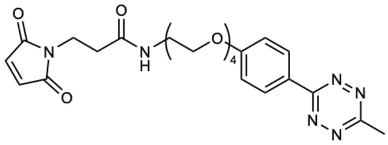	516.6

Protein modification

LCMS

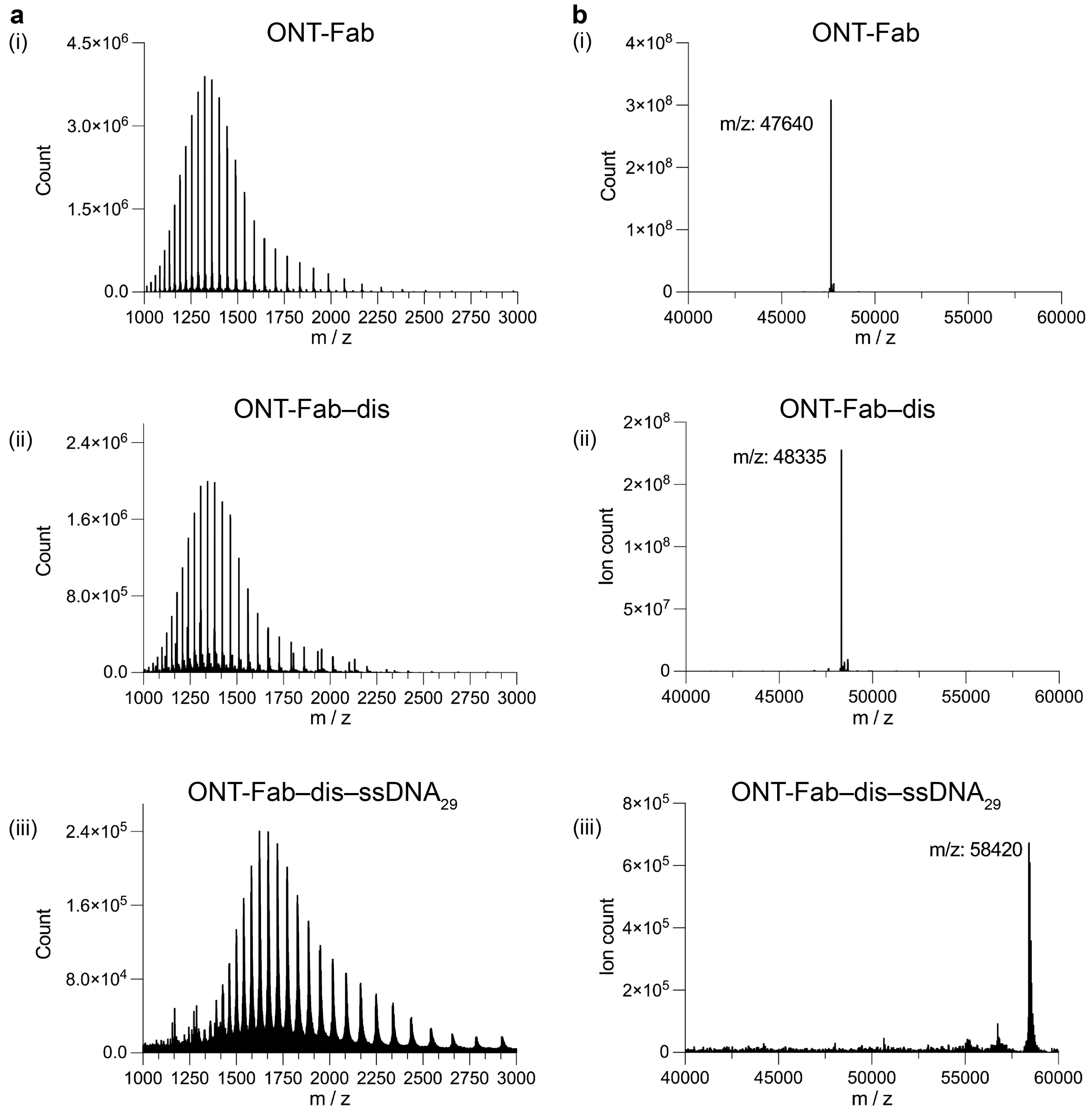

Figure S2. Successful conjugation of ssDNA$_{29}$ to ONT-F(ab) can be confirmed using LCMS. **a**, Raw ion traces and **b** deconvoluted mass spectra for (i) ONT-F(ab), (ii) ONT-F(ab)–ds, and (iii) ONT-F(ab)–ds–ssDNA$_{29}$. Clean peak-to-peak conversions were observed at each conjugation stage.

DNA Modification

Sequences

Table S2. Sequences, theoretical molecular masses, and predicted extinction coefficients for the ssDNA oligonucleotides used in this study. Theoretical molecular masses were calculated from the molecular formulas. Extinction coefficients were calculated from the sequences.

Name	Sequence (N_3–5'– 3')	Theoretical molecular mass (g × mol^{-1})	Predicted extinction coefficient e_{260} (M^{-1} × cm^{-1})
$ssDNA_6$	ATC AGC	2066.2	67000
$ssDNA_{10}$	TCG GAT GGA C	3358.0	112000
$ssDNA_{15}$	GGA AGT CGC TGG GAA	4972.1	178200
$ssDNA_{20}$	CAG CGC GTC CTA TAT CGG AG	6408.0	215200
$ssDNA_{29}$	ACC TTA ATA CGA CTC ACT ATA GGG TCT CC	9086.7	313800
$ssDNA_{40}$	GAA TTA GTA GAG TGC CGC TTT GAG CCC CCC TGT CGT CGC T	12544.9	416600
$ssDNA_{50}$	CTA AGA TGC TGG ACA CTG GGT AAA GTT AAT GCG GCT GCT CTG GTC TAA GG	15811.0	565000

LCMS

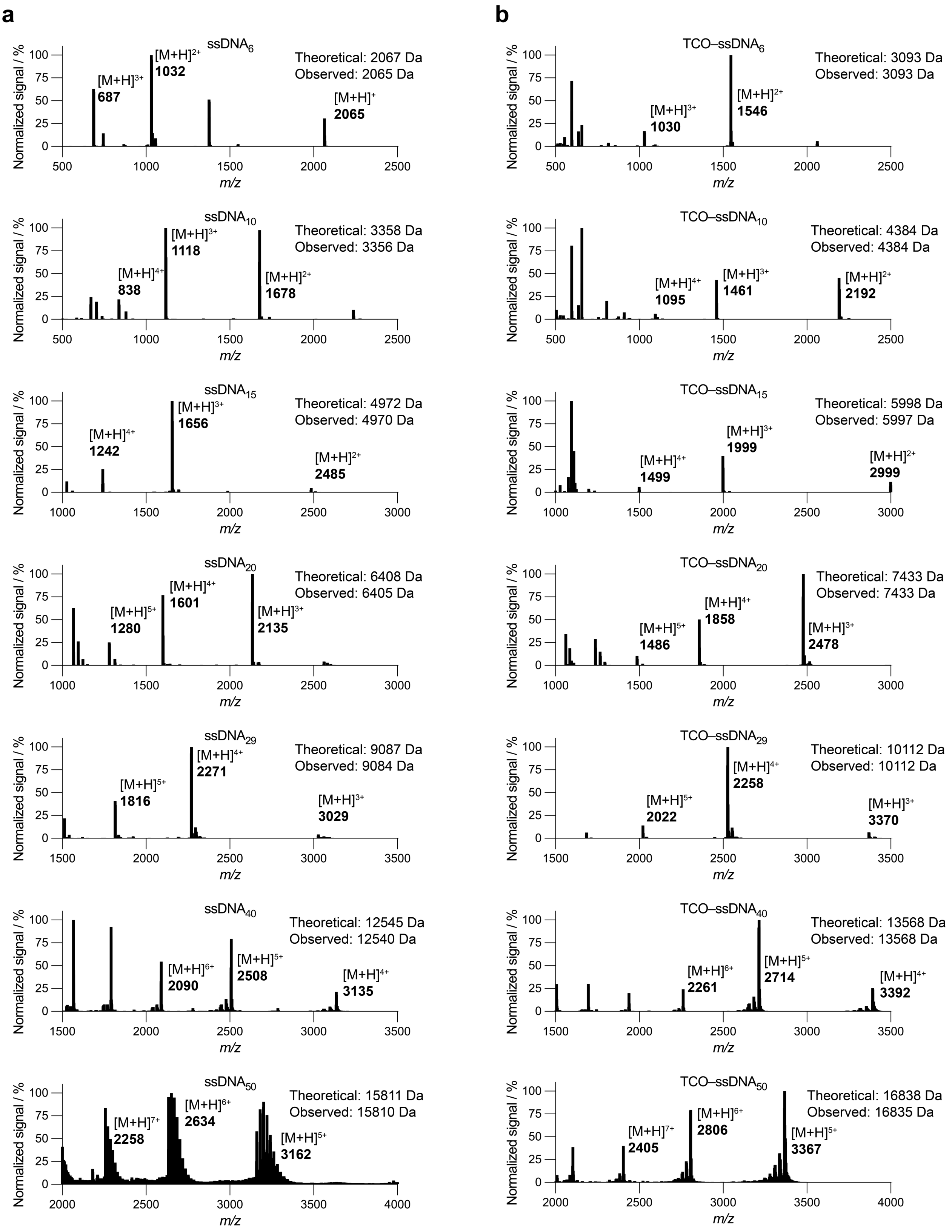

Fig. S3. TCO groups can be easily installed onto azide-containing oligonucleotides. LCMS ion traces for the native **(a)** and modified **(b)** oligonucleotides used in this study. The predicted molecular mass for each TCO functionalised oligonucleotide was calculated as the sum of the theoretical molecular mass of the starting material + the mass of the TCO linker. The observed molecular mass for each TCO functionalised oligonucleotide was calculated from the *m/z* values, accounting for the charge states.

Protein–ssDNA conjugation

Optimisation of ONT-F(ab)–ssDNA$_{29}$ conjugation

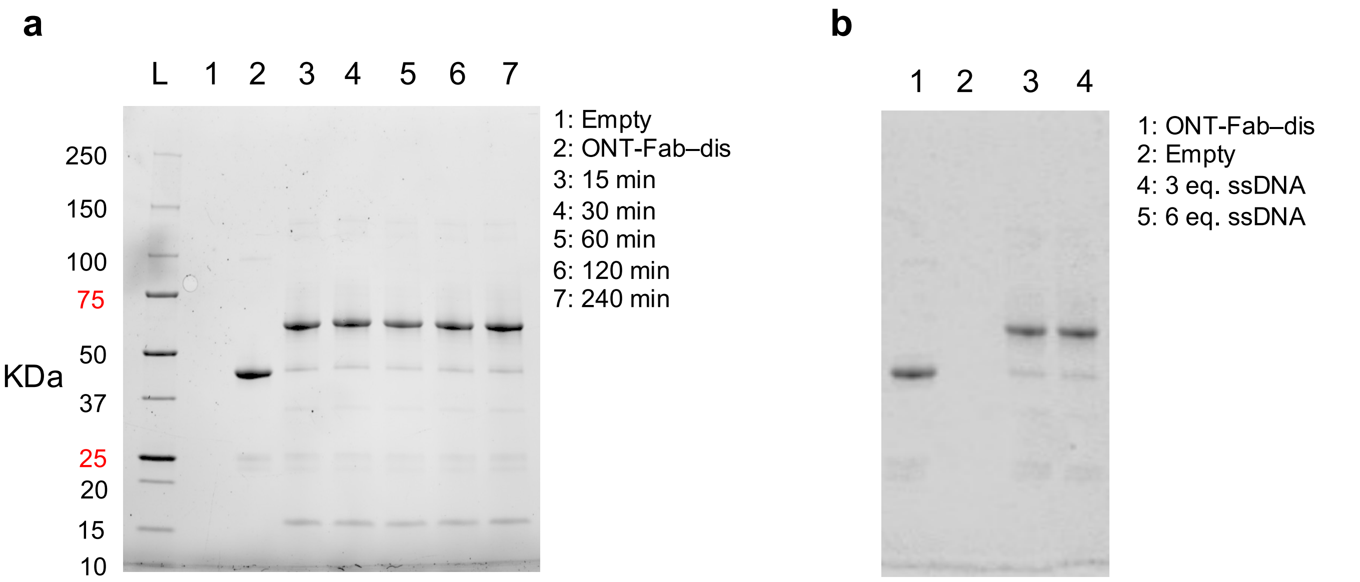

Figure S4. The reaction between ONT-F(ab)–dis and TCO–ssDNA$_{29}$ is highly efficient. **a**, The reaction with three equivalents of ssDNA$_{29}$–TCO, monitored over time. **b**, The reaction as a function of equivalents of TCO–ssDNA$_{29}$. The reaction occurs within 15 minutes using as little as 3 eq. of the TCO–ssDNA$_{29}$.

Densitometry and UV/Vis spectrometry analysis

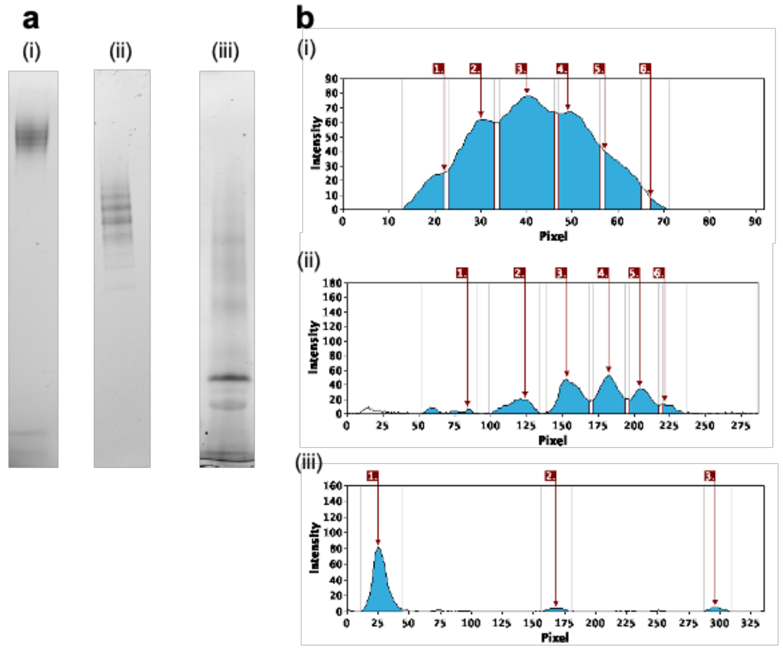

Fig. S5. Non-specific conjugation to lysine residues leads to heterogeneous products. **a**, SDS-PAGE analysis of (i) ONT–lys–ssDNA$_{29}$, (ii) ONT-F(ab)–lys–ssDNA$_{29}$, and (iii) ADAPT6–lys–ssDNA$_{29}$. **b**, Densitometry plots obtained from **a** (i), (ii), and (iii). Analysis yielded average ssDNA:protein ratios of 3.2:1, 3.5:1, and 1.16:1 for ONT–lys–ssDNA$_{29}$, ONT-F(ab)–lys–ssDNA$_{29}$, and ADAPT6–lys–ssDNA$_{29}$, respectively. The plots were generated using GelAnalyzer version 19.1.

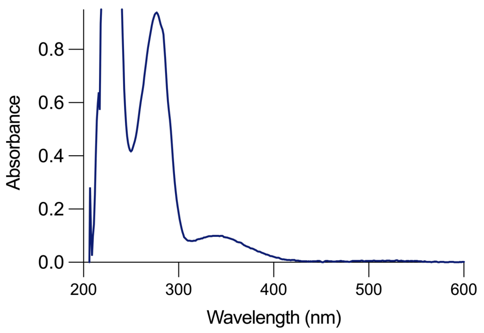

Figure S6. UV-Vis spectrum for ONT–dis. The peak at 335 nm is characteristic of the pyridazinedione and suggests a pyridazinedione:antibody ratio of 3.3 (see equation 2, main text).[3]

Decreasing the lysine labelling of ONT-Fab

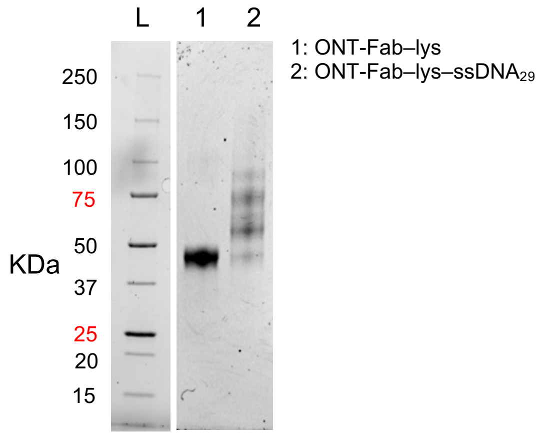

Figure S7. SDS-PAGE analysis of the conjugation between ONT-Fab–lys and TCO–ssDNA$_{29}$, with an average ssDNA:protein ratio of 1.35 : 1. ONT-Fab–lys was incubated with 4. equiv of ssDNA$_{29}$–TCO. A visible band just below 50 Kda indicates the presence of leftover ONT-Fab–lys. The ladder was run on a different gel, as indicated by the white gap.

SPR analysis

Determining MTL effects

The equations in this section were obtained from Chapter 2 of the book *Surface Plasmon Resonance, Methods and Protocols*.[7]

Mass transport limitations (MTL) in SPR arise when the rate of association between the analyte and ligand (reaction flux) is faster than the rate of diffusion of the analyte to the surface-bound ligand (transport flux). In these situations, the association between analyte and ligand becomes diffusion limited, and the observed association kinetics are no longer representative of the ligand–analyte interaction being studied. Dissociation rates are also affected in this case, as the high association rate implies that the dissociated analyte can rebind to the surface faster than it can diffuse into the bulk. Therefore, in order to validate the calculated k_{on} and k_{off}, it is essential to exclude the presence of mass transport effects during SPR experiments.

The extent of MTL in a system is given by equation 1:

$$MTL = \frac{L_r}{L_r + L_m} \qquad (1)$$

Where L_m is the transport coefficient, which describes the reaction flux, and L_r is the Onsager coefficient, which describes the transport flux. In totally diffusion-limited systems MTL is equal to 1, and as the number approaches 0 the effects of diffusion become negligible. L_m can be calculated directly from the mass transport rate k_{tr}, according to equation 2.

$$L_m = k_{tr} \cdot \frac{10^{-9}}{MW} \qquad (2)$$

For 1:1 interactions, k_{tr} can be obtained by global kinetic analysis of the SPR curves using equations 3 and 4.

$$A_0 \underset{k_{tr}}{\overset{k_{tr}}{\rightleftharpoons}} A$$
$$A + B \underset{k_{off}}{\overset{k_{on}}{\rightleftharpoons}} AB \qquad (3, 4)$$

Here, A_0 denotes the analyte in the bulk, A the analyte at the surface of the sensor chip, and B the immobilised ligand. Thus, k_{tr} denotes the rate of transport from the bulk solution to the sensor surface.

The Onsanger coefficient L_r is given by equation 5.

$$L_\mathrm{r} = k_\mathrm{on} \cdot [B] \qquad (5)$$

Where k_{on} is the association rate obtained from the global kinetic analysis described in equations 3 and 4. Notably, k_{on} must be expressed in $m^3 \cdot mol^{-1} \cdot s^{-1}$, and [B] in $mol \cdot m^{-2}$. Conversion of [B] in RU to $mol \cdot m^{-2}$ can be achieved using equation 6.

$$[B]_{\mathrm{RU}} = R_{max} \cdot \frac{10^{-6}}{\mathrm{MW}} \tag{6}$$

Values for k_{tr}, L_m, L_r, and *MTL* for each of the affinity proteins we studied can be found in Tables SX and SX. In summary, we observed no significant MTL effects

SPR data summary tables

Table S3. Summarised values for k_{on}, k_{off}, K_D, and R_{maxO}:R_{maxT} obtained from the SPR plots in Fig. 2 and equation 1.

		Native (i)	dis/cys modified (ii)	dis/cys–ssDNA (iii)	lys modified (iv)	lys–ssDNA (v)
ONT	K_{on} / M^{-1} . s^{-1}	7.90 × 10^5	5.80 × 10^5	3.20 × 10^5	6.40 × 10^5	2.0 × 10^5
	K_{off} / s^{-1}	4.00 × 10^{-5}	3.80 × 10^{-5}	3.5 × 10^{-5}	4.30 × 10^{-5}	3.5 × 10^{-5}
	K_D / nM	0.051	0.066	0.11	0.067	0.18
	R_{maxO}:R_{maxT}	0.51	0.51	0.33	0.49	0.27
ONT-Fab	K_{on} / M^{-1} . s^{-1}	3.30 × 10^5	3.10 × 10^5	2.30 × 10^5	1.00 × 10^5	0.13 × 10^5
	K_{off} / s^{-1}	1.80 × 10^{-4}	2.00 × 10^{-4}	1.80 × 10^{-4}	1.90 × 10^{-4}	1.50 × 10^{-4}
	K_D / nM	0.55	0.65	0.78	1.90	12
	R_{maxO}:R_{maxT}	0.54	0.53	0.46	0.47	0.20
ADAPT6	K_{on} / M^{-1} . s^{-1}	1.50 × 10^{-5}	0.44 × 10^{-5}	0.23 × 10^{-5}	0.17 × 10^{-5}	N/A
	K_{off} / s^{-1}	5.3 × 10^{-4}	5.2 × 10^{-4}	4.9 × 10^{-4}	90 × 10^{-4}	N/A
	K_D / nM	3.5	12	21	53	N/A
	R_{maxO}:R_{maxT}	0.7	0.64	0.34	0.38	N/A

Table S4. Theoretical R_{max} and R_{maxO}:R_{maxT} calculations for ONT, ONT-F(ab), and ADAPT6, along with their respective bioconjugates. The molecular weight of each ligand was calculated using the respective molecular weights of the native ligands, the linkers, and TCO–$ssDNA_{29}$. Protein:ssDNA ratios, as obtained from densitometry or UV-Vis spectrometry analysis, were taken into account.

Ligand	Molecular weight / kDa	HER2 surface coating / RU	Theoretical R_{max} (R_{maxT}) / RU	Observed R_{max} (R_{maxO}) / RU	R_{maxO}: R_{maxT}
ONT	145165	18	37	19	0.51
ONT–dis	146626	18	37	19	0.51
ONT–dis–$ssDNA_{29}$	164743	18	42	14	0.33
ONT–lys	146776	18	37	18	0.49
ONT–lys–$ssDNA_{29}$	179045	18	45	12	0.27
ONT-Fab	47640	210	141	76	0.54
ONT-Fab–dis	48336	210	143	76	0.53
ONT-Fab–dis–$ssDNA_{29}$	58420	210	173	80	0.46
ONT-Fab–lys	49402	210	146	68	0.47
ONT-Fab–lys–$ssDNA_{29}$	84697	210	251	49	0.20
ADAPT6	7335	800	83	58	0.7
ADAPT6–cys	7850	800	88	56	0.64
ADAPT6–cys–$ssDNA_{29}$	17934	800	202	68	0.34
ADAPT6–lys	7919	800	89	33	0.38
ADAPT6–lys–$ssDNA_{29}$	19616	800	221	N/A	N/A

Table S5. Transport rate constants (k_{tr}), transport coefficients (L_m), and Onsager coefficients (L_r) for the affinity protein–ssDNA conjugates studied in Fig, 2. These values were calculated from the molecular weight, R_{maxO}, and association constants (k_{on}), as described above.

Ligand	Molecular weight / Da	R_{maxO} / mol . m^{-2}	k_{tr} / RU . M^{-1} . s^{-1}	k_{on} / mol^{-1} . m^3 . s^{-1}	L_m / m . s^{-1}	L_r / m . s^{-1}	MTL
ONT	145165	1.34×10^{-10}	1.83×10^{9}	816	1.26×10^{-5}	1.10×10^{-7}	8.62×10^{-3}
ONT–dis	146626	1.29×10^{-10}	2.22×10^{9}	590	1.51×10^{-5}	7.61×10^{-8}	5.00×10^{-3}
ONT–dis–ssDNA$_{29}$	164743	8.68×10^{-11}	1.12×10^{9}	321	6.80×10^{-6}	2.79×10^{-8}	4.08×10^{-3}
ONT–lys	146776	1.21×10^{-10}	6.54×10^{9}	650	4.46×10^{-5}	7.88×10^{-8}	1.77×10^{-3}
ONT–lys–ssDNA$_{29}$	179045	6.87×10^{-11}	3.00×10^{11}	205	1.68×10^{-3}	1.41×10^{-8}	8.40×10^{-6}
ONT-Fab	47640	1.60×10^{-9}	6.22×10^{10}	329	1.31×10^{-3}	1.88×10^{-7}	1.44×10^{-4}
ONT-Fab–dis	48336	1.47×10^{-9}	5.05×10^{13}	327	1.04	4.81×10^{-7}	4.61×10^{-7}
ONT-Fab–dis–ssDNA$_{29}$	58420	1.22×10^{-9}	1.09×10^{9}	118	1.87×10^{-5}	1.32×10^{-7}	7.04×10^{-3}
ONT-Fab–lys	49402	1.40×10^{-9}	7.46×10^{8}	106	1.51×10^{-5}	1.48×10^{-7}	9.70×10^{-3}
ONT-Fab–lys–ssDNA$_{29}$	84697	6.02×10^{-10}	2.12×10^{6}	16.9	2.5×10^{-8}	1.02×10^{-8}	2.89×10^{-1}
ADAPT6	7335	7.95×10^{-9}	1.22×10^{9}	150	1.66×10^{-4}	1.19×10^{-6}	7.12×10^{-3}
ADAPT6–cys	7850	7.19×10^{-9}	6.28×10^{8}	42.8	8.00×10^{-5}	3.08×10^{-7}	3.83×10^{-3}
ADAPT6–cys–ssDNA	17934	3.71×10^{-9}	3.45×10^{7}	24.5	1.92×10^{-6}	9.10×10^{-8}	4.52×10^{-2}
ADAPT6–lys	7919	4.46×10^{-9}	2.51×10^{8}	36	3.17×10^{-5}	1.60×10^{-7}	5.04×10^{-3}
ADAPT6–lys–ssDNA$_{29}$	19616	N/A	N/A	N/A	N/A	N/A	N/A

Table S6. Summarised values for k_{on}, k_{off}, K_D, and R_{maxO}:R_{maxT} obtained from the SPR plots in Fig. 3 and equation 1.

	ONT-Fab–dis	ONT-Fab–dis–ssDNA$_6$	ONT-Fab–dis–ssDNA$_{10}$	ONT-Fab–dis–ssDNA$_{15}$	ONT-Fab–dis–ssDNA$_{20}$	ONT-Fab–dis–ssDNA$_{29}$	ONT-Fab–dis–ssDNA$_{40}$	ONT-Fab–dis–ssDNA$_{50}$
K_{on} / M^{-1} . s^{-1}	3.20×10^5	2.40×10^5	2.10×10^5	1.60×10^5	1.40×10^5	1.20×10^5	1.30×10^5	1.20×10^5
K_{off} / s^{-1}	2.0×10^{-4}	1.90×10^{-4}	1.90×10^{-4}	1.80×10^{-4}	1.7×10^{-4}	1.90×10^{-4}	1.90×10^{-4}	1.90×10^{-4}
K_D / nM	0.63	0.79	0.90	1.1	1.2	1.6	1.5	1.6
R_{maxO}:R_{maxT}	0.49	0.47	0.46	0.42	0.42	0.38	0.34	0.31

Table S7. Theoretical R_{max} and R_{maxO}:R_{maxT} calculations for ONT-F(ab)-dis and ONT-F(ab)-dis–$ssDNA_{6–50}$. The molecular weight of each ligand was calculated using the respective molecular weights of the native ligands, the linkers, and TCO–$ssDNA_{29}$. Protein:ssDNA ratios, as obtained from densitometry or UV-Vis spectrometry analysis, were taken into account.

Sample	Molecular weight / kDa	HER2 surface coating / RU	Theoretical R_{max} (R_{maxT}) / RU	Observed R_{max} (R_{maxO}) / RU	R_{maxO}:R_{maxT}
ONT-Fab–dis	48336	210	143	70	0.49
ONT-Fab–dis–$ssDNA_6$	51400	210	152	72	0.47
ONT-Fab–dis–$ssDNA_{10}$	52691	210	156	71	0.46
ONT-Fab–dis–$ssDNA_{15}$	54305	210	161	68	0.42
ONT-Fab–dis–$ssDNA_{20}$	55741	210	165	70	0.42
ONT-Fab–dis–$ssDNA_{29}$	58420	210	173	65	0.38
ONT-Fab–dis–$ssDNA_{40}$	61878	210	183	63	0.34
ONT-Fab–dis–$ssDNA_{50}$	65144	210	193	60	0.31

Table S8. Transport rate constants (k_{tr}), transport coefficients (L_m), and Onsager coefficients (L_r) for the affinity protein–ssDNA conjugates studied in Fig, 3. These values were calculated from the molecular weight, R_{maxO}, and association constants (k_{on}) as described above.

Sample	Molecular weight / Da	R_{maxO} / mol . m^{-2}	k_{tr} / RU . M^{-1} . s^{-1}	k_{on} / mol^{-1} . m^3. s^{-1}	L_m / m . s^{-1}	L_r / m . s^{-1}	MTL
ONT-Fab–dis	48336	1.47×10^{-9}	5.05×10^{13}	327	1.04	4.81×10^{-7}	4.61×10^{-7}
ONT-Fab–dis–$ssDNA_6$	51400	1.40×10^{-9}	6.69×10^{9}	241	1.30×10^{-4}	3.38×10^{-7}	2.59×10^{-3}
ONT-Fab–dis–$ssDNA_{10}$	52691	1.36×10^{-9}	4.08×10^{9}	215	7.74×10^{-5}	2.93×10^{-7}	3.76×10^{-3}
ONT-Fab–dis–$ssDNA_{15}$	54305	1.25×10^{-9}	1.47×10^{9}	160	2.71×10^{-5}	2.00×10^{-7}	7.34×10^{-3}
ONT-Fab–dis–$ssDNA_{20}$	55741	1.25×10^{-9}	1.10×10^{9}	140	1.97×10^{-5}	1.75×10^{-7}	8.81×10^{-3}
ONT-Fab–dis–$ssDNA_{29}$	58420	1.12×10^{-9}	1.09×10^{9}	118	1.87×10^{-5}	1.32×10^{-7}	7.04×10^{-3}
ONT-Fab–dis–$ssDNA_{40}$	61878	1.02×10^{-9}	1.19×10^{9}	137	1.92×10^{-5}	1.39×10^{-7}	7.20×10^{-3}
ONT-Fab–dis–$ssDNA_{50}$	65144	9.18×10^{-10}	1.05×10^{9}	120	1.61×10^{-5}	1.10×10^{-7}	6.79×10^{-3}

Supporting SPR data

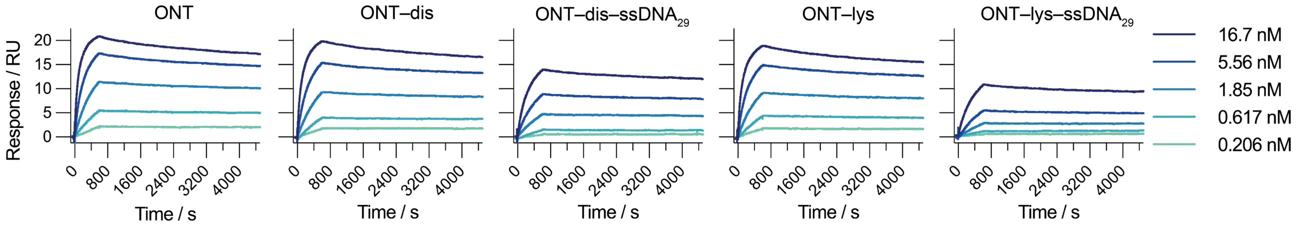

Figure S8. Extended dissociation times are required to obtain k_{off} for ONT ligands**.** SPR curves for (i) ONT, (ii) ONT-dis, (iii) ONT–dis–ssDNA29, (iv) ONT–lys, (v) ONT–lys–ssDNA29 binding to HER2 with extended dissociation times. A dissociation time of over 60 minutes was required to obtain reliable fits. Measured data (solid line) was fit to a 1:1 binding model (dashed line).

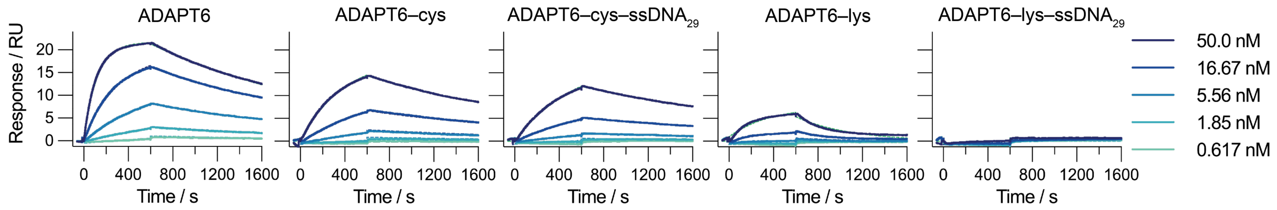

Figure S9. ADAPT6 ligands bind poorly to HER2 at low on-chip densities. SPR sensorgrams for (i) ADAPT6, (ii) ADAPT6–cys, (iii) ADAPT6–cys–ssDNA$_{29}$, (iv) ADAPT6–lys, and (v) ADAPT6–lys–ssDNA$_{29}$ binding to HER2 at low ligand density. Measured data (solid line) was fit to a 1:1 binding model (dashed line).

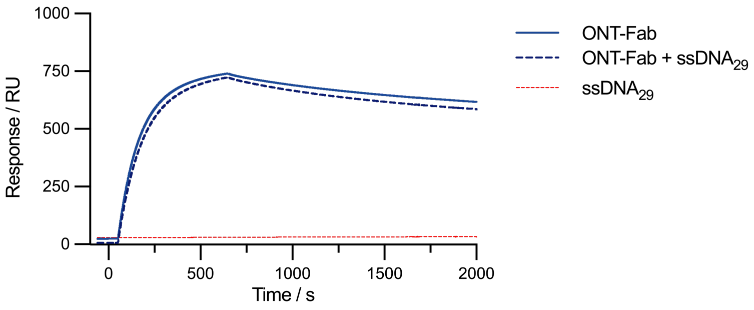

Figure S10. The presence of ssDNA$_{29}$ does not impact the binding of the ligands to the SPR chip**.** SPR sensorgrams for ONT-F(ab) (solid blue line), ssDNA$_{29}$ (red dotted line), and ONT-F(ab) + 200 nM ssDNA$_{29}$ (green dashed line). These controls demonstrate that ssDNA$_{29}$ does not bind to HER2 and that the presence of ssDNA$_{29}$ (unconjugated) does not impact the binding of ONT-F(ab). Measured data (solid line) was fit to a 1:1 binding model (dashed line).

ImmunoPCR

SDS-PAGE

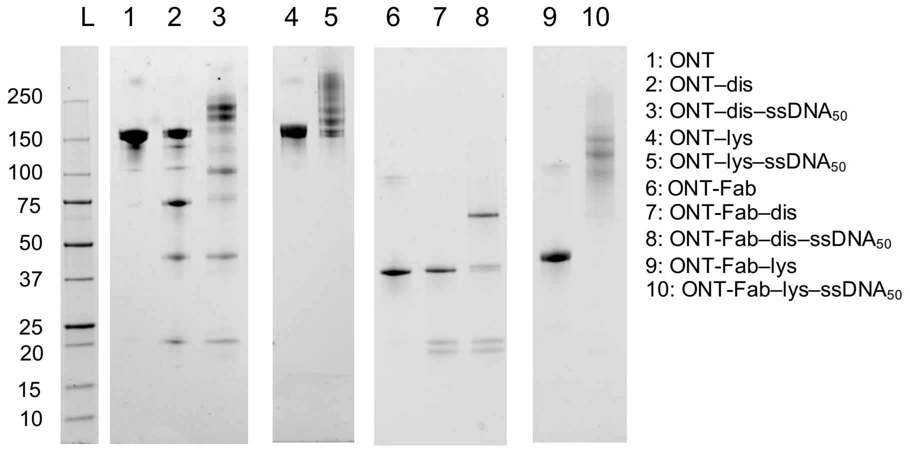

Figure S11. The optimised conjugation protocol is transferable to larger ssDNA oligonucleotides. SDS-PAGE analysis of the modified ONT and ONT-Fab conjugates employed for the immuno-PCR assay (Fig. 4). Once again, more homogenous products are obtained using site-selective approaches (lanes 3 and 8).

qPCR

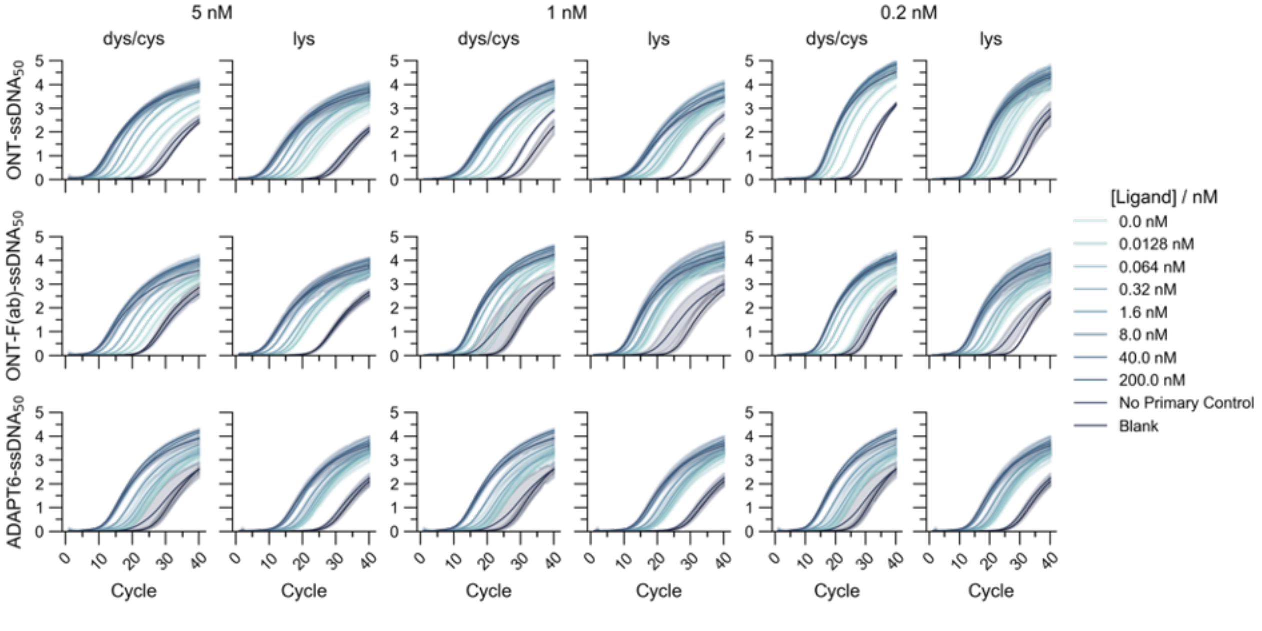

Figure S12. qPCR curves obtained for the immuno-PCR assays of HER2 titrated against ONT–$ssDNA_{pcr}$ and ONT-Fab–$ssDNA_{pcr}$. The probes were evaluated at 5, 1, and 0.2 nM. The solid curves and shaded areas represent the mean and standard deviation values of three measurements.

Cytometry and cell staining

SDS-PAGE

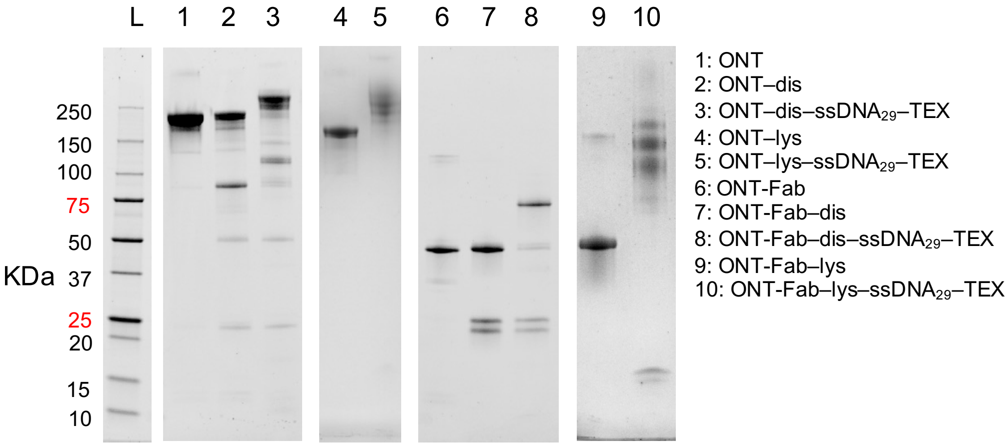

Figure S13. The optimised conjugation protocol is transferable to fluorescently tagged ssDNA oligonucleotides. SDS-PAGE analysis of the modified ONT and ONT-Fab conjugates employed for the flow cytometry and fluorescence microscopy (Fig. 5). As before, more homogenous products are obtained using site-selective approaches (lanes 3 and 8).

Contour plots

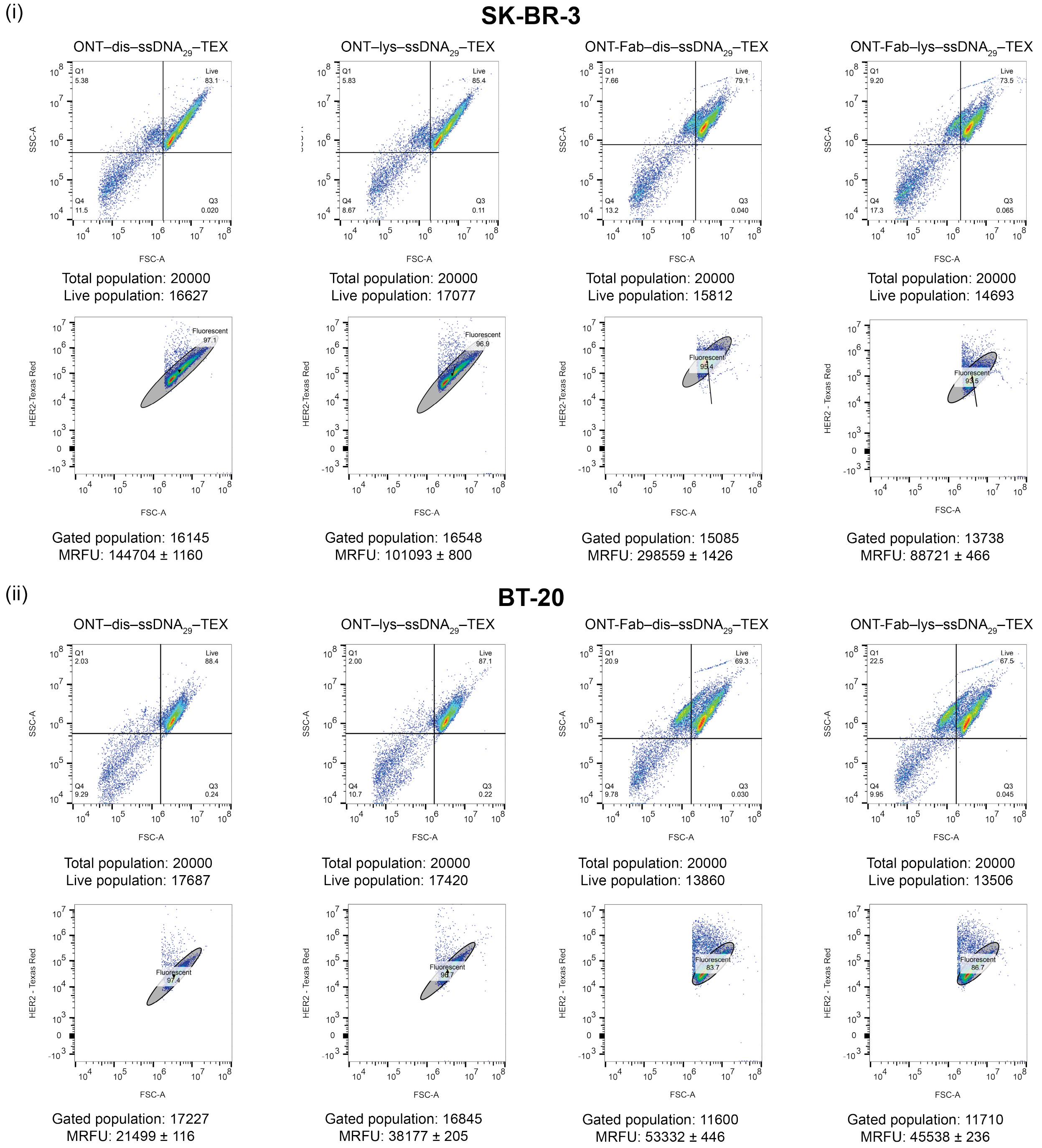

Figure S14. Contour plots for the cytometry data presented in Fig. 5a. Contour plots and gating for (i) SK-BR-3 and (ii) BT-20 cells stained with 10 nM of ONT–dis–ssDNA29–TEX, ONT–lys–ssDNA29–TEX, ONT-Fab–dis–ssDNA29–TEX, and ONT-Fab–lys–ssDNA29–TEX. Quadrants were defined for the ONT and ONT-Fab ligands, with live cells contained within quadrant 2. The percentage of live cells for each ligand is reported at the top right of each contour plot. Fluorescently-stained cells were gated from the population using a single ellipsoid of defined area. Magnetic gating was employed, with movement based around the centre of the ellipsoid. The arrow represents the magmetic movement of the ellipsoid. Mean relative fluorescence (MRFU) values are reported as the mean ± SEM.

Alternative Gating

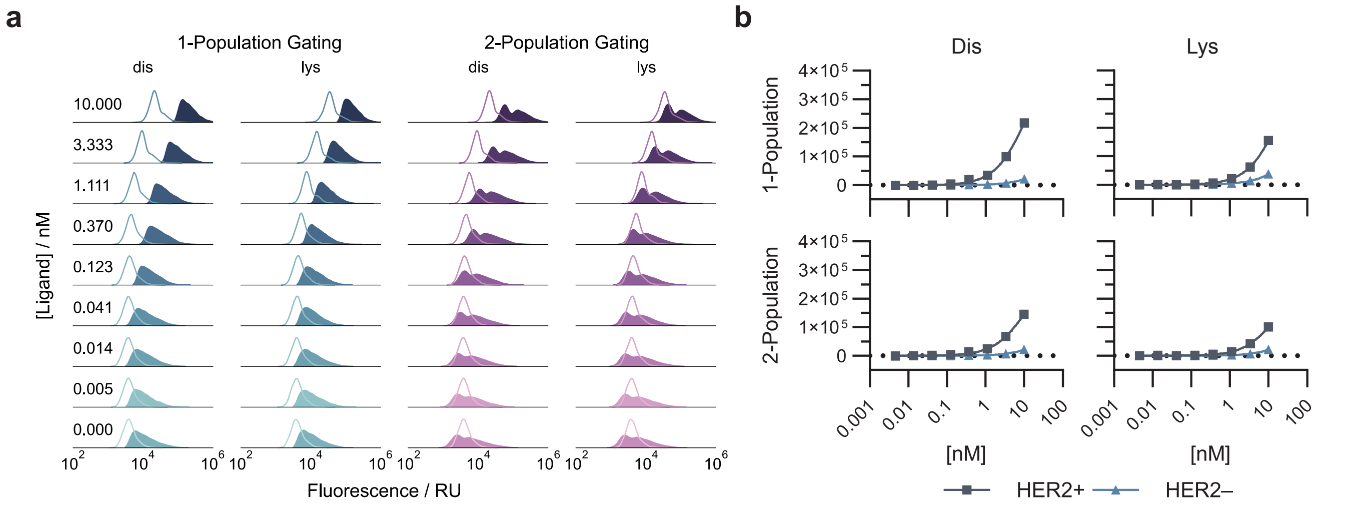

Figure S15. Comparison of normalised fluorescence signal distribution functions of SK-BR-3 (HER2 positive, blue filled) and BT-20 (HER2 negative, clear lines) cells stained with varying concentrations of ONT–dis–ssDNA$_{29}$–TEX, ONT–lys–ssDNA$_{29}$–TEX, ONT-Fab–dis–ssDNA$_{29}$–TEX, and ONT-Fab–lys–ssDNA$_{29}$–TEX, using an alternative gating approach to select for a single cell population versus the gating employed in the main text. The resulting observed trend is unaffected.

Control experiments

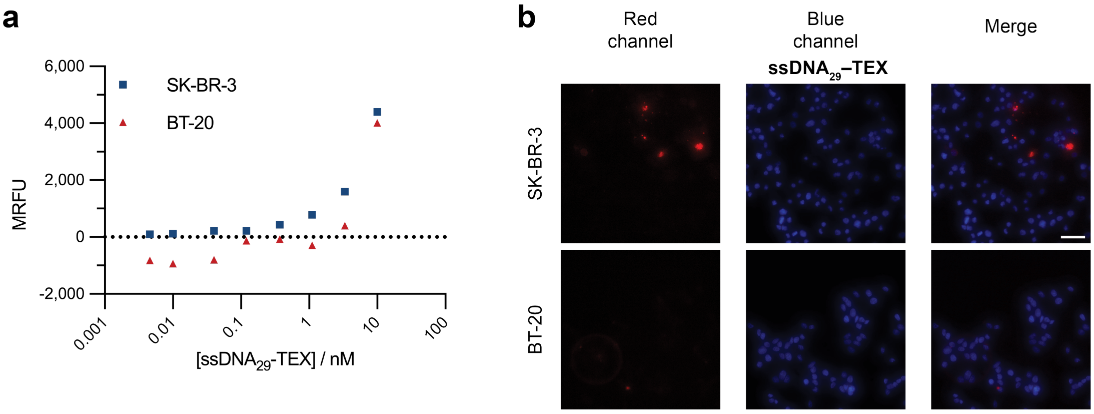

Figure S16. Non-specific binding between the HER2+ and HER- cells and ssDNA$_{29}$–TEX is not significantly different. **a,** Mean fluorescence value vs ligand concentration for SK-BR-3 (HER2+, blue squares) and BT-20 (HER2-, red triangles) cells stained with ssDNA$_{29}$–TEX as determined by flow cytometry. Mean fluorescence values were obtained from histograms comprising 10,000 - 20,000 measurements. Comparison with the values obtained for staining of SK-BR-3 cells with the DNA conjugated ligands (Fig. 5b) shows a 25-50 fold decrease in signal. **b,** Fluorescence microscopy images showing staining of SK-BR-3 and BT-20 cells using ssDNA$_{29}$–TEX. Cells were stained with DAPI after fixing. The cells were imaged under 40x magnification using red (E_{550}/Em_{630}) and blue (E_{377}/Em_{442}) filter sets a 100 ms exposure, and laser power (SpectraX-6-LCR) at 50%. The data clearly show low binding between ssDNA$_{29}$–TEX and both the SK-BR-3 and BT-20 cells.

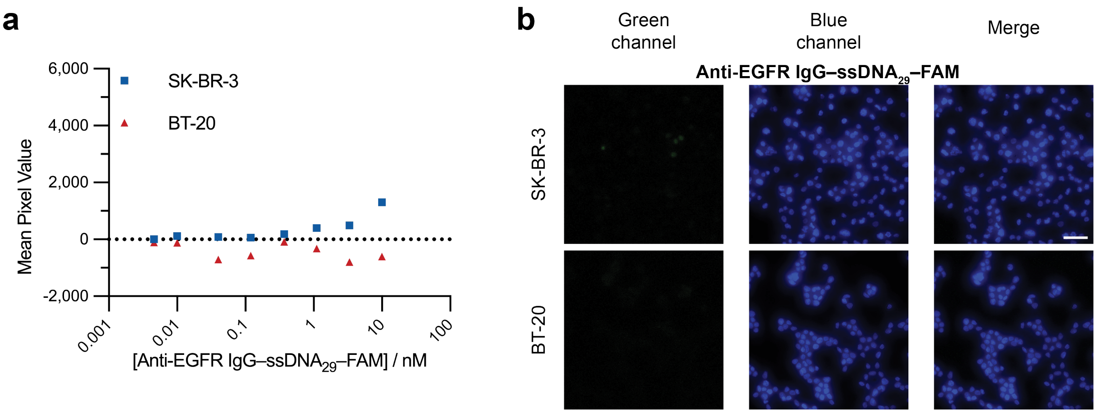

Figure S17. Non-specific binding between the HER2+ and HER- cells and an anti-EGFR (HER1) IgG–ssDNA$_{29}$ conjugate is not significantly different. **a,** Mean fluorescence value vs ligand concentration for SK-BR-3 (HER2+, blue squares) and BT-20 (HER2-, red triangles) cells stained with Anti-EGFR IgG–ssDNA$_{29}$–FAM as determined by flow cytometry. Mean fluorescence values were obtained from histograms comprising 10,000 - 20,000 measurements. **b,** Fluorescence microscopy images showing staining of SK-BR-3 and BT-20 cells using Anti-EGFR IgG–ssDNA$_{29}$–FAM. Cells were stained with DAPI after fixing. The cells were imaged under 40x magnification using green (E_{478}/Em_{525}) and blue (E_{377}/Em_{442}) filter sets, a 100 ms exposure, and laser power (SpectraX-6-LCR) at 50%. The data clearly show low binding between Anti-EGFR IgG–ssDNA$_{29}$–FAM and both the SK-BR-3 and BT-20 cells. Anti-EGFR IgG–ssDNA$_{29}$–FAM was prepared using the protocol for non-specific lysine conjugation.

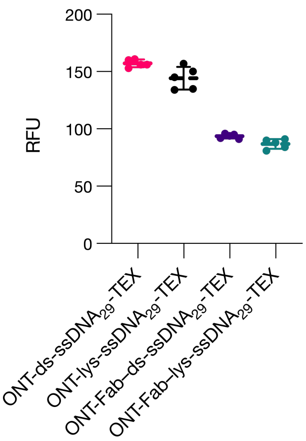

Figure S18. Fluorescence differences between site-selectively and non-selectively modified ligands are not significant**.** Red fluorescence values (Ex_{550}/Em_{630}) (in PBS) of each of the ligands used in the cell-membrane staining studies. Dotted lines represent the mean of the five plotted values, and the error bars represent the standard deviation. No significant differences were observed between the two conjugation strategies.

References

[1] J. Nilvebrant, M. Åstrand, M. Georgieva-Kotseva, M. Björnmalm, J. Löfblom, S. Hober, *PLoS One* **2014**, *9*, e103094.

[2] M. Greene, D. A. Richards, J. Nogueira, K. Campbell, P. Smyth, M. Fernandez, C. J. Scott, V. Chudasama, *Chem. Sci.* **2017**, *9*, 79–87.

[3] V. Chudasama, M. E. B. Smith, F. F. Schumacher, D. Papaioannou, G. Waksman, J. R. Baker, S. Caddick, *Chemical Communications* **2011**, *47*, 8781–8783.

[4] A. D. Edelstein, M. A. Tsuchida, N. Amodaj, H. Pinkard, R. D. Vale, N. Stuurman, *J Biol Methods* **2014**, *1*, e10.

[5] J. Schindelin, I. Arganda-Carreras, E. Frise, V. Kaynig, M. Longair, T. Pietzsch, S. Preibisch, C. Rueden, S. Saalfeld, B. Schmid, J. Y. Tinevez, D. J. White, V. Hartenstein, K. Eliceiri, P. Tomancak, A. Cardona, *Nature Methods* **2012**, *9*, 676–682.

[6] D. A. Richards, M. R. Thomas, P. A. Szijj, J. Foote, Y. Chen, J. C. F. Nogueira, V. Chudasama, M. M. Stevens, *Nanoscale* **2021**, *13*, 11921–11931.

[7] J. M. Walker, *Surface Plasmon Resonance, Methods and Protocols*, **n.d.**
