# Supplementary material for: Site-directed conjugation of single-stranded DNA to affinity proteins: quantifying the importance of conjugation strategy
Source: Chem Sci. 2024 May 7;15(23):8982–92. doi: 10.1039/d4sc01838a (PMC11168188; doi:10.1039/d4sc01838a)
Supplement: SC-015-D4SC01838A-s002 [file SC-015-D4SC01838A-s002.pdf]

Electronic Supplementary Material (ESI) for Chemical Science.
This journal is © The Royal Society of Chemistry 2024

List of Figures

Protein–ssDNA Conjugation **G1**

Main Text Figure 1 G1
SI Figure S4 Panel A G1
SI Figure S4 Panel B G1
SI Figure S5 G2
SI Figure S7 G2

The impact of DNA length on HER2 binding **G3**

Main Text Figure 3 G3

ImmunoPCR **G3**

SI Figure S11 G3

Cytometry and cell staining **G4**

SI Figure S13 G4

Protein–ssDNA Conjugation

Main Text Figure 1

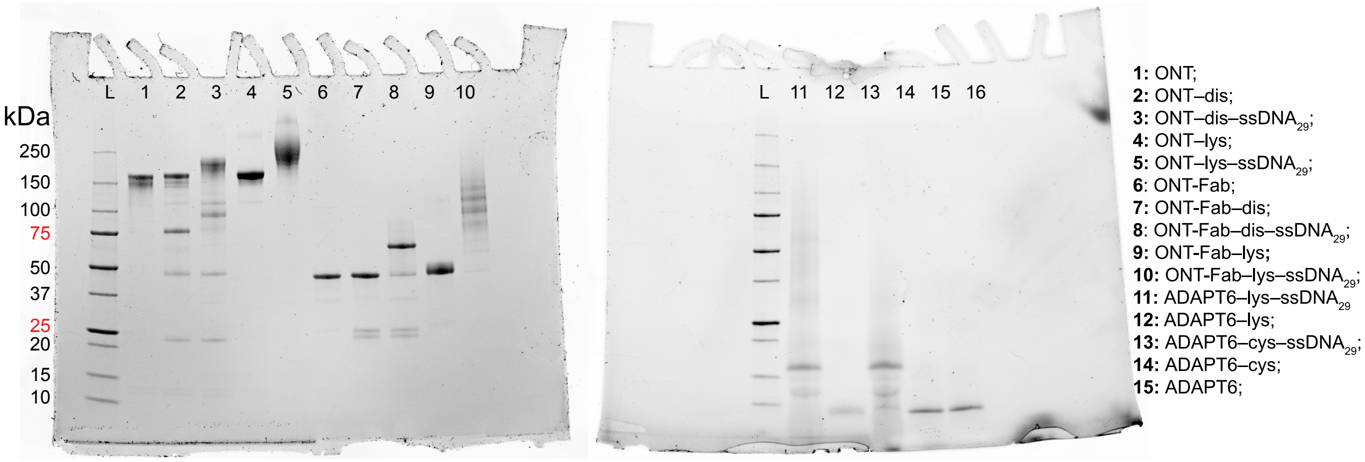

Figure G1. Un-cropped SDS-PAGE images for the analysis of ONT, ONT-Fab and ADAPT6 modified structures.

SI Figure S4 Panel A

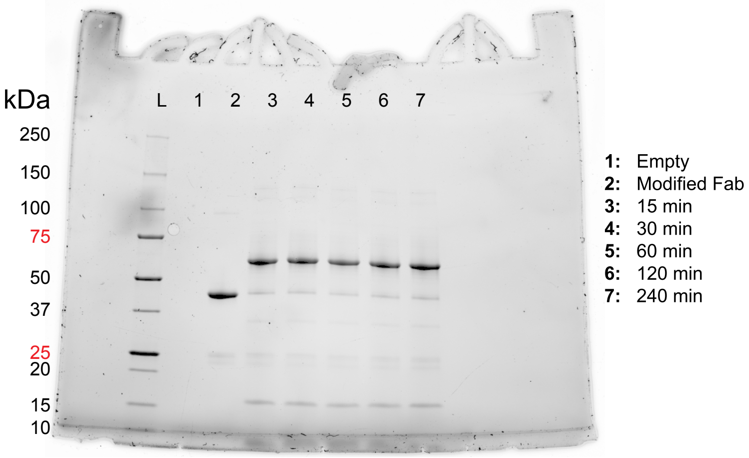

Figure G2. Un-cropped SDS-PAGE image for the analysis of the reaction between ONT-Fab–dis and TCO–ssDNA$_{29}$, monitored over time.

SI Figure S4 Panel B

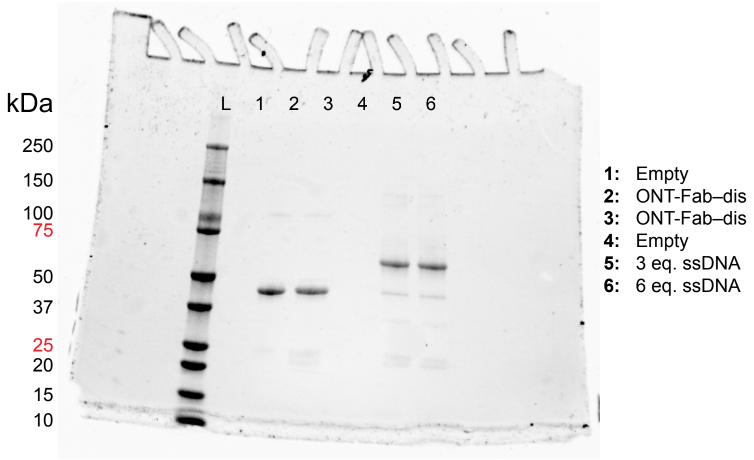

Figure G3. Un-cropped SDS-PAGE image for the analysis of the reaction between ONT-Fab–dis and TCO–ssDNA$_{29}$, as a function of equivalents of ssDNA.

SI Figure S5

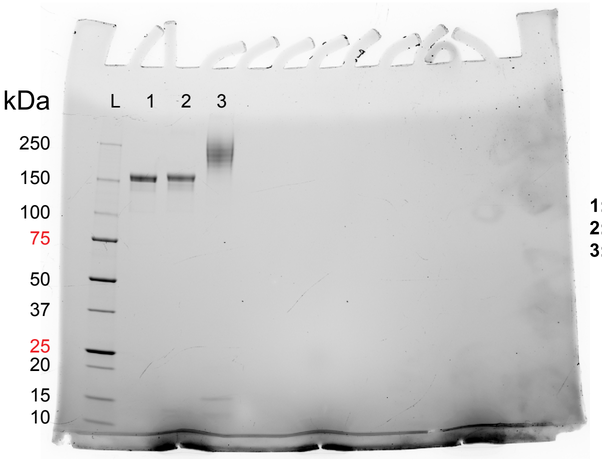

1: ONT;
2: ONT–lys;
3: ONT–lys–ssDNA$_{29}$;

Figure G4. Un-cropped SDS-PAGE image(s) for the analysis of the heterogeneity of the non-specific conjugation to lysisne residues. Specificaly, image shows panel (i) in original figure. Panels (ii) and (ii) were extracted from images in Figure G1 from lanes 10 and 11 respectively.

SI Figure S7

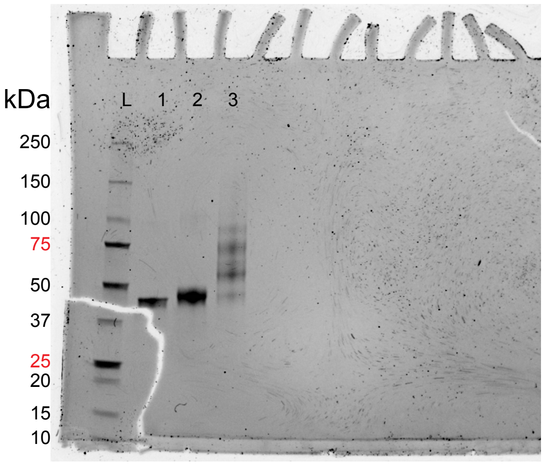

1: ONT-Fab;
2: ONT-Fab–lys;
3: ONT-Fab–lys–ssDNA$_{29}$;

Figure G5. Un-cropped SDS-PAGE image for the analysis of the conjugation between ONT-Fab–lys and TCO–ssDNA$_{29}$, with an average ssDNA:protein ratio of 1.35 : 1.

The impact of DNA length on HER2 binding

Main Text Figure 3

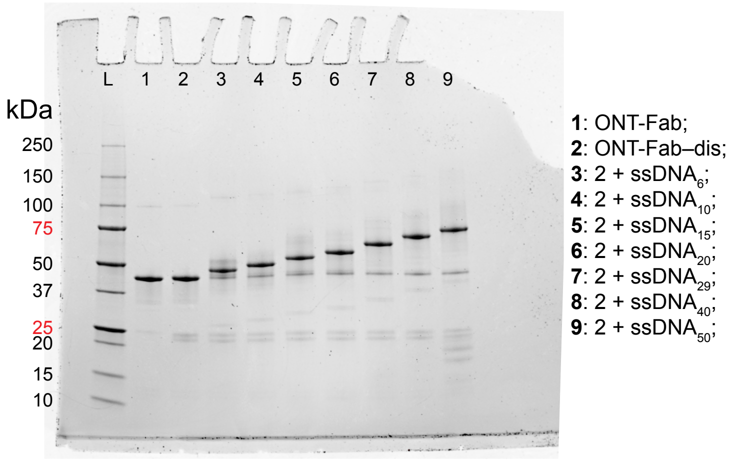

Figure G6. Un-cropped SDS-PAGE image for the analysis of ONT-Fab–$ssDNA_{6–50}$ conjugates.

ImmunoPCR

SI Figure S11

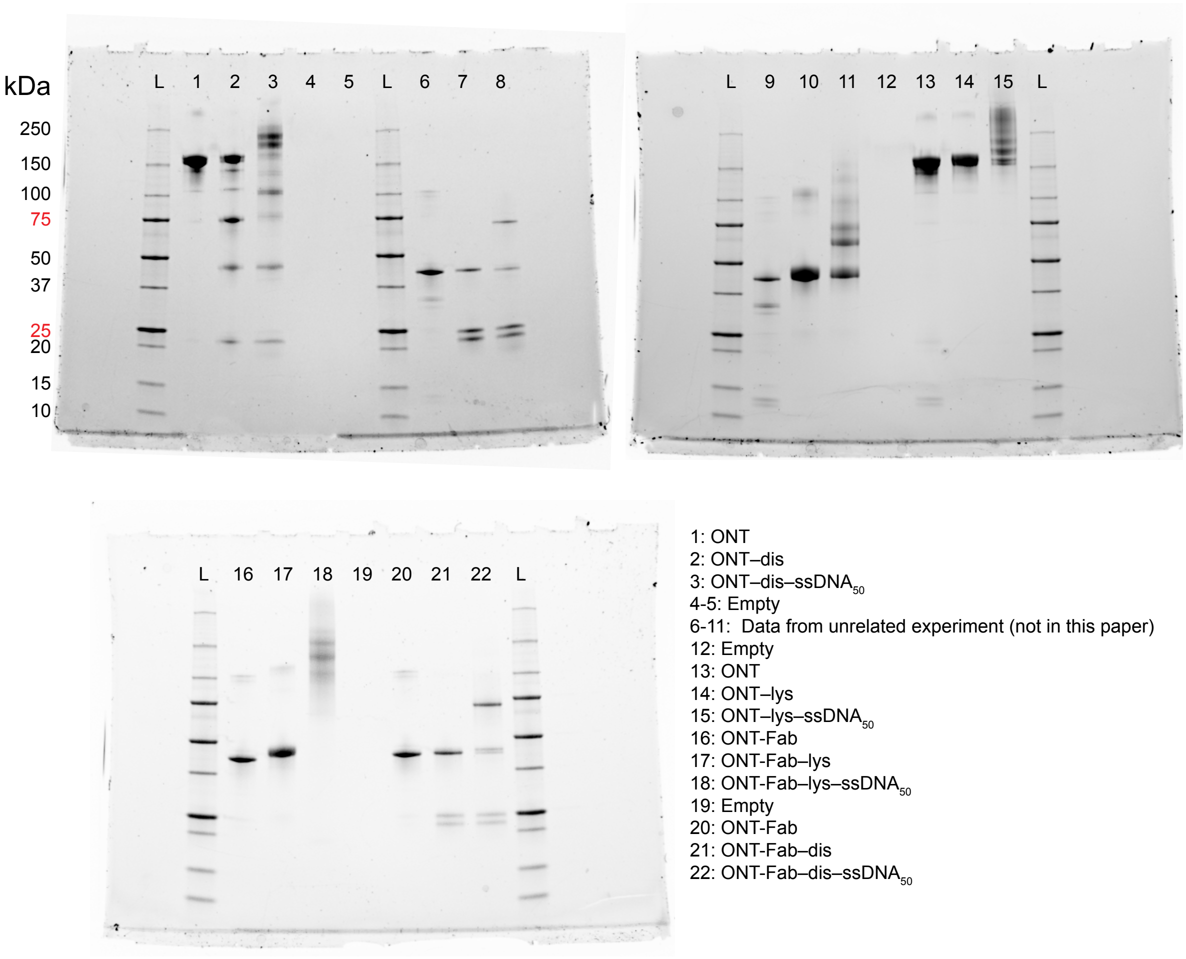

Figure G7. Un-cropped SDS-PAGE images for the analysis of Protein–$ssDNA_{50}$ conjugates for the immunoPCR experiments.

Cytometry and cell staining

SI Figure S13

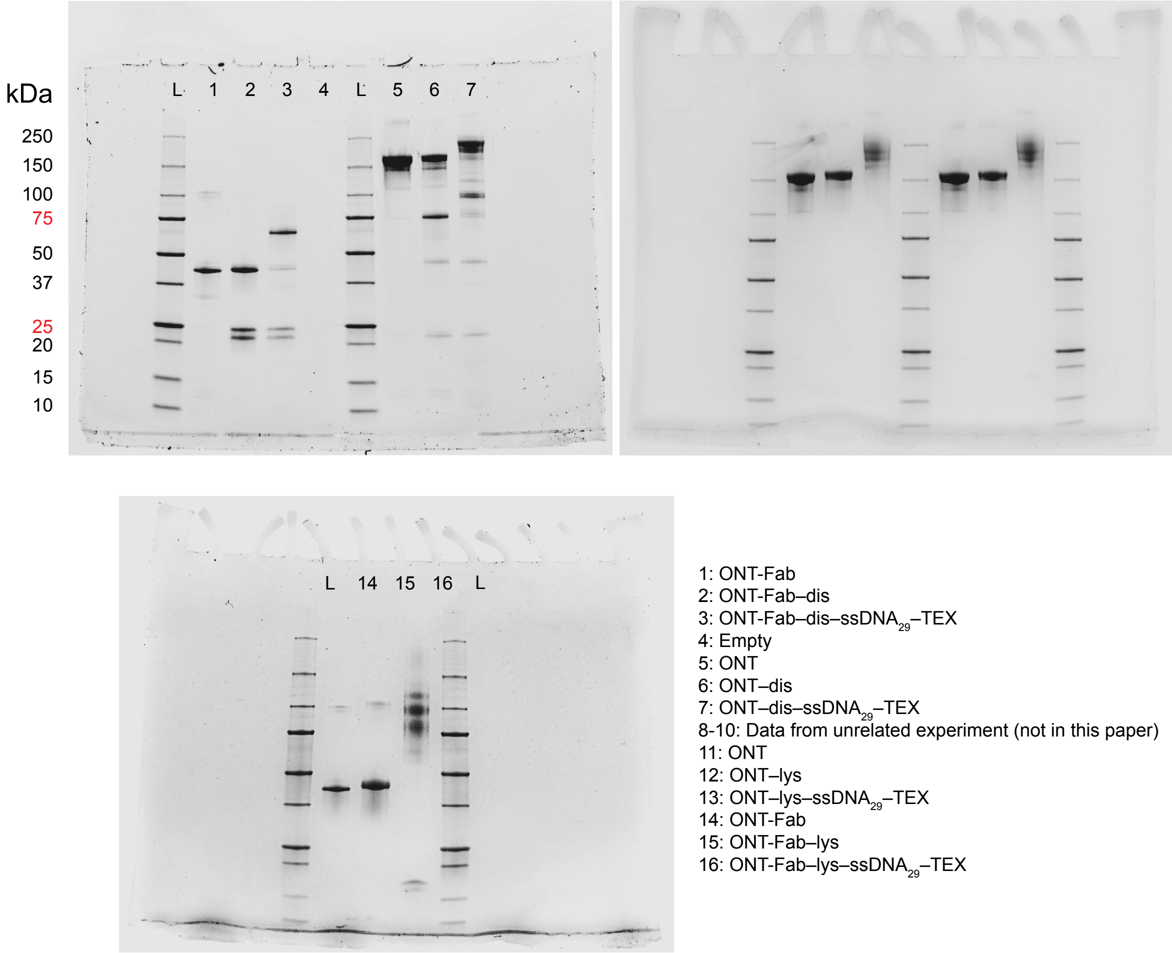

Figure G8. Un-cropped SDS-PAGE images for the analysis of Protein–ssDNA$_{29}$–TEX conjugates for the cell flow cytometry and imaging experiments.
